# Sea ice presence is linked to higher carbon export and vertical microbial connectivity in the Eurasian Arctic Ocean

**DOI:** 10.1038/s42003-021-02776-w

**Published:** 2021-11-03

**Authors:** Eduard Fadeev, Andreas Rogge, Simon Ramondenc, Eva-Maria Nöthig, Claudia Wekerle, Christina Bienhold, Ian Salter, Anya M. Waite, Laura Hehemann, Antje Boetius, Morten H. Iversen

**Affiliations:** 1 Alfred Wegener Institute, Helmholtz Center for Polar and Marine Research, D-27570 Bremerhaven, Germany; 2 Max Planck Institute for Marine Microbiology, D-28359 Bremen, Germany; 3 Institute for Ecosystem Research, Kiel University, D-24118 Kiel, Germany; 4 Faroe Marine Research Institute, FO 100 Tórshavn, Faroe Islands; 5 Ocean Frontier Institute, NS, B3H 4R2 Halifax, Canada; 6 MARUM and University of Bremen, D-28359 Bremen, Germany; 7 Present Address: Department of Functional and Evolutionary Ecology, University of Vienna, A-1090 Vienna, Austria

**Keywords:** Biogeochemistry, Microbial biooceanography

## Abstract

Arctic Ocean sea ice cover is shrinking due to warming. Long-term sediment trap data shows higher export efficiency of particulate organic carbon in regions with seasonal sea ice compared to regions without sea ice. To investigate this sea-ice enhanced export, we compared how different early summer phytoplankton communities in seasonally ice-free and ice-covered regions of the Fram Strait affect carbon export and vertical dispersal of microbes. In situ collected aggregates revealed two-fold higher carbon export of diatom-rich aggregates in ice-covered regions, compared to *Phaeocystis* aggregates in the ice-free region. Using microbial source tracking, we found that ice-covered regions were also associated with more surface-born microbial clades exported to the deep sea. Taken together, our results showed that ice-covered regions are responsible for high export efficiency and provide strong vertical microbial connectivity. Therefore, continuous sea-ice loss may decrease the vertical export efficiency, and thus the pelagic-benthic coupling, with potential repercussions for Arctic deep-sea ecosystems.

## Abstract (web summary)

Fadeev et al. explore carbon export dynamics along the water column using microscopic analysis, 16S rRNA gene amplicon sequencing, and physical modeling of data from long-term sediment traps in the Fram Strait. Their results indicate that larger aggregates from sea-ice and under-ice diatom blooms are responsible for higher export efficiency and vertical microbial connectivity, suggesting that continuous sea-ice loss may result in decreased pelagic-benthic coupling, with resultant impacts on marine food webs.

## Introduction

The Arctic Ocean is currently undergoing unprecedented changes due to advancing climate warming. Ice coverage is declining by ~13% per decade compared to the mean September extent for 1981–2010^1^, and climate models project that business-as-usual scenarios will result in seasonally ice-free conditions by 2050^2^. Increasing temperature, combined with declining sea ice extent, ice thickness and multiyear ice^3^ are impacting the composition of primary producers in the Arctic Ocean^4^. For example, Atlantic phytoplankton species, such as *Phaeocystis* spp., are already seasonally reoccurring in the Fram Strait^5^, and were also recently observed during a phytoplankton bloom in the central Arctic Ocean^6^. It has been suggested that temperate phytoplankton species will become resident in the Eurasian Basin of the Arctic Ocean if the intrusion of warming Atlantic waters continues^7^. As primary producers form the base of the food web, such shifts are likely to have drastic consequences, not just in the pelagic realm, but also for pelagic-benthic coupling and biogeochemical cycling in the Arctic Ocean^8^. However, the complexity of factors driving Arctic productivity regionally makes it difficult to generalize to the future carbon flux in the entire Arctic Ocean^9^.

Remote sensing of ocean color in the Arctic shelf seas suggests an increase in net primary production by 57% since 1998^10^, which likely enhances vertical carbon and nutrient fluxes^11^. Furthermore, the transformation from thick multiyear to thin first-year sea ice is increasing light transmission through it^12^. Accordingly, field observations show an increased spatial and temporal extent of sea-ice algae and under-ice pelagic phytoplankton in the Arctic basins, for which ocean color based primary production assessments are not available^6,13–15^. When sea ice melts, such ice-algae and under-ice phytoplankton blooms that are mostly composed of diatoms can also deliver substantial pulses of carbon and nutrients to benthic ecosystems^8,16,17^. For example in 2012, the release of fast-sinking ice-algae, the *Melosiraarctica*, from melting sea ice delivered up to 9 g of carbon per square meter of seafloor, which was more than 85% of the total carbon export that year^16^. Primary production models suggest that with the northward propagation of ice-edge blooms along leads and polynyas, the impact of ice-algae and under-ice phytoplankton blooms on productivity is likely to increase^18^, with ecological consequences for pelagic and benthic ecosystems^16,19^. However, it remains unclear what ecological effects an earlier and a stronger retreat of the ice-edge will have for carbon export on the Arctic shelf seas and margins. Key factors could be the timing of stratification, e.g. by meltwater, the phytoplankton composition during the bloom, mismatches to grazers, and other effects on carbon export efficiency, including the microbial loop in the euphotic zone and remineralization of aggregates during sinking to the deep ocean.

It is well established that sinking particles are an essential conduit of carbon and nutrients to heterotrophic organisms in the deep ocean^20^. The microbial loop retains most of the carbon and the nutrients in the surface ocean, and can influence carbon export efficiency substantially, next to other factors such as particle size and activity of grazers^21^. Recent studies have also revealed that sinking particles function as connecting vectors that disperse microbes between the surface and the deep ocean, some of which may carry specific heterotrophic functions in the remineralization of sinking matter^22–25^. Hence, beyond the supply of energy and nutrients, sinking particles can play a key role in determining the structure and functioning of microbial communities in the deep sea by importing microbes from surface waters^22,23,25^. To date, such so-called vertical microbial connectivity has mainly been demonstrated in temperate and tropical oceanic settings^22–27^. However, Rapp et al. ^28^ found that in Central Arctic sea-ice algae which reached the seafloor influenced its microbial community composition. Considering the changes that the Arctic Ocean is currently undergoing, it is critical to understand how carbon export will be impacted by changing sea-ice regimes and associated alterations in the composition and rates of primary producers. Potential changes in carbon export may also affect vertical microbial connectivity through the water column. With increasing evidence of functional microbial groups exported from surface waters into the deep sea, alterations in vertical microbial connectivity may impact biogeochemical processes of the deep ocean and the seafloor.

**Fig. 1:**
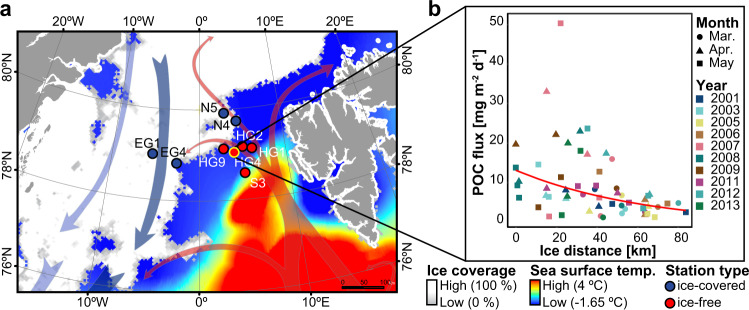
Overview of sampling area in the Fram Strait. **a** Monthly average of sea ice cover and sea surface temperature during July 2016. The arrows represent general directions of the West Spitzbergen Current (in red) and the East Greenland Current (in blue). The yellow edge of the HG4 station indicates the position of a long-term sediment trap deployment. **b** Particulate Organic Carbon (POC) flux collected at 200 m by a long-term moored sediment trap between 2001 and 2013 during the early spring (March-May), plotted as a function of the distance to the ice-edge. The regression shows that there was a significantly negative relationship between the distance to the ice-edge and the magnitude of POC flux (*y* = 13.6 × e^−0.015 × *x*^), *R*^2^ = 0.2, *p* < 0.001).

Here, we assess the efficiency of particle export and vertical connectivity in the Fram Strait under seasonally ice-covered and ice-free conditions, which were defined based on temporal duration of sea ice presence during the productive season. The Fram Strait represents the major deep-water gateway to the Arctic Ocean basins. Warm Atlantic-water flows northwards via the West Spitzbergen Current through the eastern part of Fram Strait, whereas cold Arctic water and sea ice flows southwards into the Atlantic via the East Greenland Current in its western part (Fig. 1a). The annual sea ice volume export through the western Fram Strait is currently increasing by 11% per decade during spring and summer due to ice thinning and increasing drift speed in and out of the Central Arctic^29^. If Atlantic warming of the Arctic Ocean continues, it is projected that stronger ice-melt will occur in the western Eurasian Basin, eventually reducing ice export through Fram Strait^30^. Here we studied the effect of sea ice distribution on phytoplankton community composition, carbon export efficiency and vertical microbial connectivity in the Fram Strait. We test the hypotheses (1) that settling aggregates formed in ice-covered regions sink faster than those formed in ice-free regions, which may result in lower export flux for ice-free regions. With regard to the effect on microbial community structure we postulated that (2) particles are colonized by free-living microbes in surface waters and have little colonization at depth, (3) that there is stronger vertical microbial connectivity in ice-covered compared to ice-free regions, and (4) that the stronger microbial connectivity leads to higher representation of surface-born microbes in the deep sea and the sediment. We found large and fast-settling diatom aggregates in ice-covered regions that led to higher carbon export efficiency near the sea ice, as well as stronger vertical microbial connectivity, compared to the seasonally ice-free region of the Fram Strait, which was dominated by smaller, slow-sinking *Phaeocystis* spp. aggregates. Our results suggest that with the ice-edge seasonally retreating from the Eurasian Basin, carbon export efficiency and vertical connectivity may decline in large regions of the Arctic Ocean.

## Results

### Settling aggregates in ice-free and ice-covered regions

We studied microbial communities associated with settling aggregates in contrasting sea ice conditions between June 24th and July 16th 2016 at the Long-Term Ecological Research (LTER) observatory HAUSGARTEN in the Fram Strait (expedition PS99.2 with RV Polarstern). Based on remote sensing sea ice concentrations during June-July 2016, we classified ice concentrations >15% as ice-covered conditions and ice concentrations <15% as ice-free conditions. According to this classification, the stations in the West Spitsbergen Current (‘HG’ and ‘S’; seafloor depths ~2500 m) were seasonally ice-free, as the majority of the productive season in 2016 (March–July) they had no sea ice (Fig. 1a; Table 1). On the other hand, in the East Greenland Current (‘EG’; seafloor depth: ~1000–2700 m) and the northern (‘N’; seafloor depth: ~2500–2800 m) stations, sea ice was present during most of the productive season, and these sites were thus defined as ice-covered stations (Fig. 1a; Table 1).

**Table 1:** Sinking aggregate trajectories characteristics of particles reaching the surface ocean between March 1st 2016 and July 31st 2016 in different regions of Fram Strait.

		Ice-covered (EG)	Ice-covered (N)	Ice-free (HG)
Station coordinates		78.81° N / 2.729° W	79.74° N / 4.185° E	79.06° N / 4.51° E
Starting depth (m) of trajectory calculation		2350	2350	1950
Number of days during 2016 with ice-coverage > 15%		197	107	78
Number of days during March-July 2016 with ice-coverage > 15%		92	38	29
Measured in situ	Sinking velocity (m d^−1^)	52	52	29
	Median catchment radius (km)	78 ± 45	74 ± 53	118 ± 97
	Aggregates originated from ice-covered waters (% of total)	72	44	16
	Median sinking trajectory length (km)	194 ± 62	181 ± 64	392 ± 111
Hypothetical low	Sinking velocity (m d^−1^)	20	20	20
	Median catchment radius (km)	142 ± 79	132 ± 115	124 ± 124
	Aggregates originated from ice-covered waters (% of total)	50	36	15
	Median sinking trajectory length (km)	536 ± 124	527 ± 159	572 ± 175
Hypothetical high	Sinking velocity (m d^−1^)	60	60	60
	Median catchment radius (km)	70 ± 46	70 ± 53	94 ± 76
	Aggregates originated from ice-covered waters (% of total)	74	41	6
	Median sinking trajectory length (km)	179 ± 55	161 ± 63	233 ± 71

The sinking trajectories were modeled using the measured in situ aggregates sinking velocities in each region, as well as using hypothetical low (20 m d^−1^) and high (60 m d^−1^) velocities. The values after ± represent standard deviation.

Particulate organic carbon (POC) fluxes at 200 m depth in the seasonally ice-free HG4 station (79.01°N, 4.20°E) were collected using a long-term moored sediment trap between 2001 and 2013. The POC fluxes showed peaks (>10 mg m^−2^ d^−1^) early in the productive season (March, April, and May), during the period when sea ice is present in the region and hence defined as ice-associated carbon export, and again later in the season (June, July, and August) during the ice-free period due to POC export of pelagic production^31^. During the ice-associated flux peak (March, April and May), the POC export between 2001 and 2013 showed an inverse relationship to the distance from the ice edge that was present at up to 86 km from the trap (Fig. 1b). However, during the summer flux peak (June, July and August), we did not observe a relationship between the POC export and the ice edge, which was present at up to 144 km from the trap (in summer 2004 the ice-edge was at 269 km from the trap; Supplementary Fig. 1). This suggests that higher sea-ice proximity may enhance POC flux. To further investigate the effect of sea ice on carbon export efficiency we assessed the exported organic matter at stations with contrasting sea ice conditions in the Fram Strait during summer 2016 (i.e., ice-covered stations ‘EG’ and ‘N’ vs. ice-free stations ‘HG’ and ‘S’). Furthermore, we explored the microbial communities associated with the sinking particles and compared the sinking velocities for the aggregates collected in the two regions as a proxy for the time required to sink from surface waters to the deep sea and the sediment in the contrasting ice conditions.

Due to potential horizontal displacement of particles during their sinking^32^, we first used a Lagrangian particle tracking algorithm to test whether our spatial classification scheme permitted the differentiation between ice-covered and ice-free origins of particles during the productive season of 2016. By using a time-dependent velocity field of a high-resolution ocean-sea ice model combined with constant particle sinking velocity, we were able to backtrack the surface origins of particles found at the seafloor of all three sampled regions (Supplementary Fig. 2). The backward trajectory calculations were performed using three scenarios: 1) sinking velocities that were measured for in situ collected aggregates from ice-covered and ice-free regions (52 and 29 m d^−1^, respectively); as well as hypothetical sinking velocities of 2) faster-sinking (60 m d^−1^) and 3) slower sinking particles (20 m d^−1^), which were chosen based on previous observations in the region^32^. In all three scenarios, the modeled sinking trajectories showed that the majority of particles in the ice-free region (‘HG’ stations) originated from ice-free surface waters (84–94%; Table 1), and were primarily from the Atlantic waters south of the investigated region (Supplementary Fig. 2). In the ice-covered region (‘EG’ and ‘N’ stations) 36–74% of the particles originated in ice-covered surface waters (Table 1). Thus, although some level of lateral transport was observed, the overall horizontal displacement of particles between ice-covered and ice-free regions during their sinking was low (Supplementary Fig. 2).

**Fig. 2:**
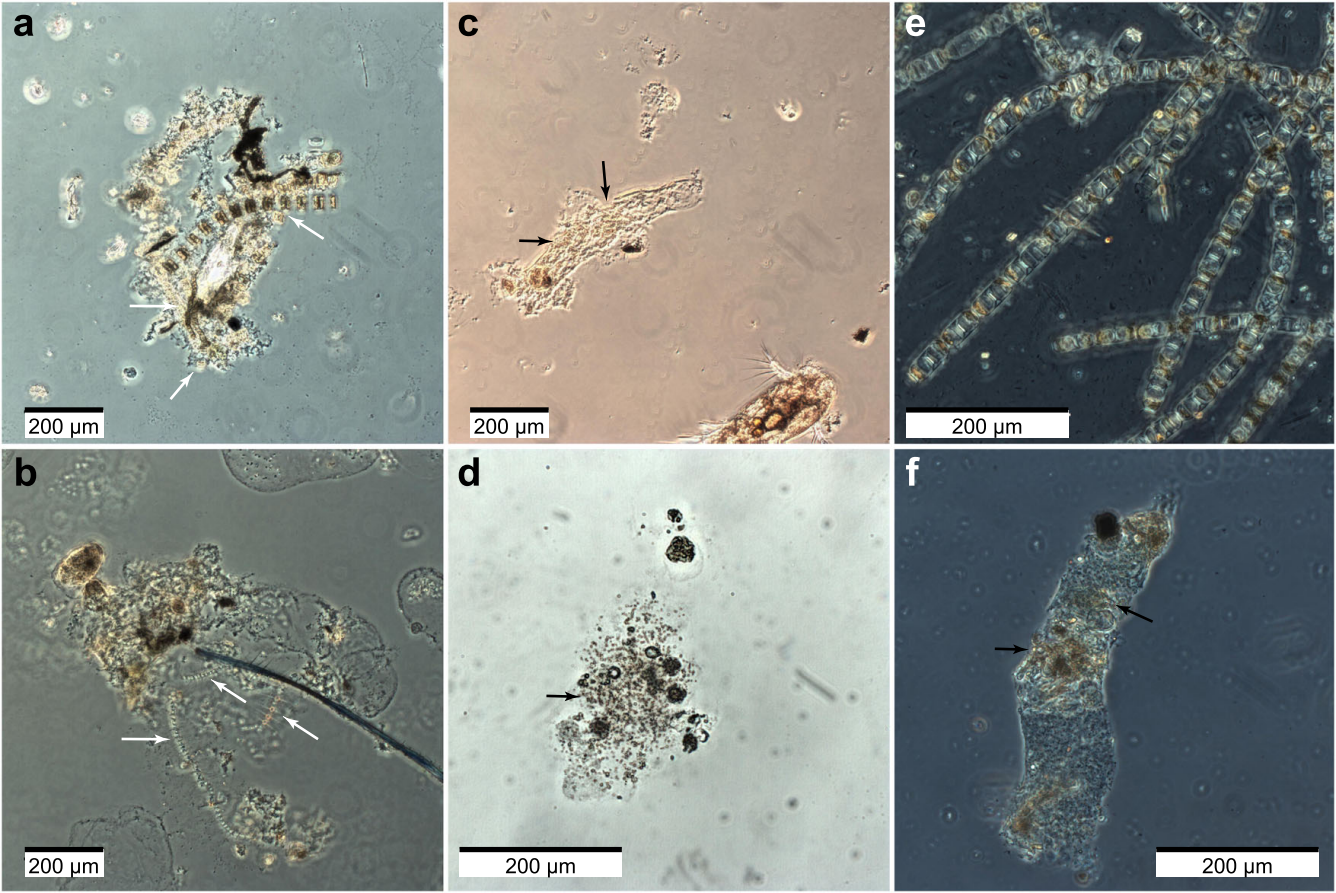
Exemplary light microscopy images of marine aggregates from MSC deployments in Fram Strait. **a***,* **b** Aggregates dominated by diatoms from ice-covered region -‘EG’, where (**a**) is mainly diatoms and (**b**) is diatoms together with *Phaeocystis* spp. **c**, **d** Aggregates dominated by flagellates in the ice-free region - ‘HG’, where (**c**) is a copepod fecal pellet composed of flagellates and (**d**) is a marine snow aggregate formed from *Phaeocystis* spp. colonies. **e** Chains of *Melosira* spp., diatoms growing under the sea ice. **f** *Calanus* spp. (copepod) fecal pellets collected at the ice-covered region - ‘N’ and formed from mainly *Phaeocystis* spp. colonies with a few diatoms. White arrows point towards diatom chains and black arrows point towards colonies of flagellates.

All sampled stations were at the later stage of the phytoplankton bloom, based on the rate of consumed nitrate, silica and phosphate above the seasonal pycnocline (50 m depth, Supplementary Data 1). When comparing the nutrient and phytoplankton concentrations between the ice-covered and ice-free stations, we did not observe any statistically significant differences (Wilcoxon Signed-Rank Test; *p* > 0.05). This suggested that both regions had similar productivity prior and during our study (Supplementary Data 1), although we cannot rule out that the two regions had different nutrient concentrations at the start of the productive season. Microscopic analyses of water samples revealed that phyto- and protozooplankton communities in the surface waters (5–10 m depth) and at chlorophyll *a* maximum (13–28 m depth) of both ice-free and ice-covered regions were dominated by *Phaeocystis* spp. and planktonic diatoms (Supplementary Data 1). Nonetheless, the ice-covered regions had much higher abundance of diatoms, compared to the ice-free region (114 ± 40 and 80 ± 27 × 10^3^ cells mL^−1^, respectively). This was further observed in the composition of in situ formed aggregates collected using a marine snow catcher (MSC) directly below the chlorophyll *a* maximum (60 m depth), where *Phaeocystis* spp. dominated aggregates of the ice-free region (total of 24 examined aggregates) and planktonic diatoms dominated the aggregates collected in the ice-covered regions (total of 36 examined aggregates; Fig. 2). The aggregates from the ice-covered regions were two-fold larger (Wilcoxon Signed-Ranks Test; *p* < 0.001; Table 2) and sank two-fold faster than the aggregates collected in the ice-free regions (Wilcoxon Signed-Rank Test; *p* < 0.05; Table 2). Half of the aggregates collected in the ice-free region (13 out of 24) were smaller than 512 µm in diameter, while almost all (33 out of 36) collected aggregates in the ice-covered regions were larger than 512 µm (Fig. 2).

**Table 2:** Vertical fluxes and marine aggregates characteristics in the epipelagic waters (0–100 m) of ice-covered and ice-free regions.

Sampling method	Characteristics	ice-covered (N)	ice-free (HG)
Drifting traps fluxes	Particulate organic carbon (POC; mg m^−2^ d^−1^)	128	63
	Particulate organic nitrogen (PON; mg m^−2^ d^−1^)	17.5	6.5
	POC to PON ratio (mol:mol)	7.9	11.3
Gel trap fluxes	Number of collected particles	2003	1399
	Total particle number flux (# m^−2^ d^−1^)	36 × 10^4^	37 × 10^4^
	Total particle volume flux (mm^3^ m^−2^ d^−1^)	11 × 10^3^	1.5 × 10^3^
	Average ESD of particles (mm)	0.2 ± 0.2 (range: 0.1–2)	0.1 ± 0.1 (range: 0.1–1)
	Average volume of particles (mm^3^)	0.03 ± 0.2	0.003 ± 0.02
Vertical flow chamber	Number of collected particles	36	24
	Average ESD of particles (mm)	0.9 ± 0.1 (range: 0.3–2.4)	0.6 ± 0.1 (range: 0.3–1.3)
	Average sinking velocity of particles (m d^−1^)	52.8 ± 0.9	29.5 ± 0.7
	Dominant phytoplankton in particles	Diatoms	Flagellates (*Phaeocystis* spp.)

*ESD* equivalent spherical diameter. The values after ± represent standard errors.

During the expedition, we deployed drifting sediment traps at 100 m depth in both the ice-free (station ‘HG’) and ice-covered regions (station ‘N’). The drifting sediment traps collected both particulate organic carbon and nitrogen flux (POC and PON, respectively), and were equipped with a viscous gel to capture and preserve the size and structure of intact settling aggregates. The gel traps confirmed the MSC observations of *Phaeocystis* spp. dominated aggregates in the ice-free regions (total of 1399 examined aggregates) and planktonic diatom-dominated aggregates in the ice-covered regions (total of 2003 examined aggregates). The gel traps showed similar numbers of particles exported in the ice-free and ice-covered regions, but confirmed that the aggregates in the ice-covered regions had on average two-fold larger diameters than those collected in the ice-free regions (Wilcoxon Signed-Ranks Test; *p* < 0.001; Table 2). The larger diameters in the ice-covered regions translated into an order of magnitude larger average volume, compared to aggregates of the ice-free regions (Table 2). Hence, the sampled larger and faster-settling aggregates in the ice-covered regions caused a two-fold higher carbon export compared to the ice-free regions (Table 2). The carbon to nitrogen ratios (C:N, mol:mol) were 11.3 in the ice-free regions and 7.9 in the ice-covered regions, indicating export of fresher material from under the sea ice (Table 2). Furthermore, at the ice-stations macroscopic strands of the sea-ice diatom *Melosira arctica* were observed by sea ice sampling (Fig. 2e), as well as during high-resolution imaging of the seafloor in the ice-covered stations (doi:10.1594/PANGAEA.873926).

### Free-living and particle-associated microbial communities

To estimate the vertical microbial connectivity in both ice-free and ice-covered regions, free-living (FL) and particle-associated (PA) microbial communities of four distinct water layers: surface (10–30 m), epipelagic (100 m), mesopelagic (1000 m) and bathypelagic (~50 m above the seafloor), were characterized using Illumina 16S rRNA gene sequencing. For the analyses of the microbial community composition we chose amplicon sequence variants (ASVs) as the highest possible taxonomic resolution that the method provides^33^. The final dataset consisted of 3,709,676 sequences from 66 samples that were assigned to 6253 ASVs associated with bacterial and archaeal lineages (Supplementary Data 2). Rarefaction curves did not reach a plateau in any of the sampled communities, however, estimated asymptotic extrapolation to double amount of sequences showed only few additional ASVs (Supplementary Fig. 3). Thus, our sequencing depth was satisfactory to represent most of the bacterial and archaeal diversity in all sampled microbial communities^34^. The classes *Alphaproteobacteria*, *Bacteroidia* and *Gammaproteobacteria* dominated the microbial communities in both FL and PA fractions, each comprised more than 15% of the sequences and more than 10% of the ASVs in the entire dataset (Supplementary Fig. 4). In the deep ocean communities (> 1000 m) there was an increasing sequence abundance of the clades SAR202 (class *Dehalococcoidia*), SAR324 (Marine group B), SAR406 (*Marinimicrobia*), and the archaeal class *Nitrososphaeria*, each comprising 1–6% of the sequences and 3–6% of the ASVs in the entire dataset (Supplementary Fig. 4).

With increasing depth in the water column, microbial communities showed an increase in both richness (based on Chao1 richness estimator; Kruskal-Wallis test; Chi square=37.24, df=3, *p* < 0.001), and diversity (based on Shannon’s diversity index; Kruskal-Wallis test; Chi square=39.89, df=3, *p* < 0.001; Supplementary Fig. 5). In the FL communities, this trend was mostly caused by significant differences between the communities of surface to epipelagic, and epi- to mesopelagic layers (post-hoc Wilcoxon Signed-Ranks Test; *p*-adjusted<0.001), with no significant differences from meso- to bathypelagic layers (post-hoc Wilcoxon Signed-Ranks Test; *p*-adjusted>0.05). In contrast, in the PA communities, the richness did not show significant changes from surface to epipelagic, and from epi- to mesopelagic layers, but significantly increased between meso- and bathypelagic layers (post-hoc Wilcoxon Signed-Ranks Test; *p*-adjusted<0.001). The diversity of the PA communities did not change significantly in the upper 100 m of the water column (i.e., from surface to epipelagic waters), but increased at meso- and bathypelagic layers (post-hoc Wilcoxon Signed-Ranks Test; *p*-adjusted=0.017; Supplementary Fig. 5).

**Fig. 3:**
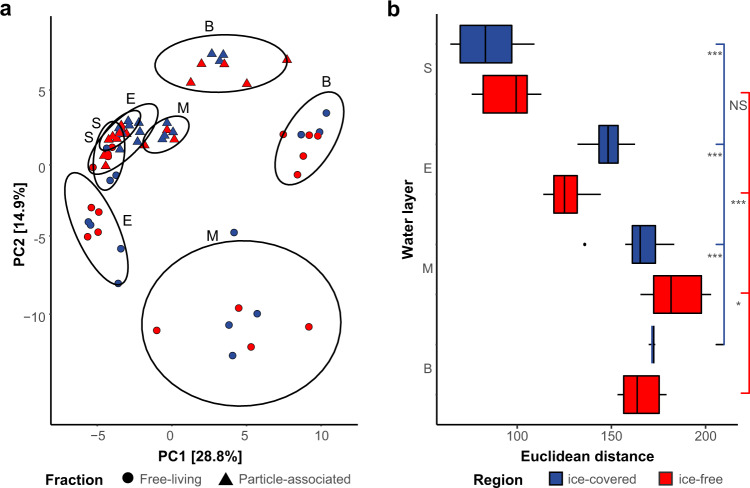
Free-living and particle-associated community patterns throughout the water column of the Fram Strait. **a** Principal component analysis (PCA) of microbial communities. Ellipses encompass clustering of each of the fractions by water layer (S-surface, E-epipelagic, M-mesopelagic, B-bathypelagic), with normal confidence of 0.95. The percentages on both axes represent the explained variance of the axis. **b** Euclidean distances between FL and PA communities in each water layer. The colors represent different geographic origins: ice-covered (blue) and ice-free (red) regions. Results of Wilcoxon signed-rank test are represented on the right edge of the figure: (NS) - not significant, *p*-adjusted > 0.05; (*) - *p*-adjusted < 0.05; (**) - *p*-adjusted < 0.01; (***) - *p*-adjusted < 0.001.

The composition of microbial communities showed a separation between the FL and PA communities along the entire water column (Fig. 3a; PERMANOVA test; *F*_*1,64*_ = 16.20, *R*^2^ = 0.11, *p* < 0.001). In both fractions the communities clustered according to the four distinct water layers (PERMANOVA test; *F*_*3,64*_ = 18.46, *R*^2^ = 0.37, *p* < 0.001). In surface and epipelagic water layers both FL and PA communities differed between the ice-covered and ice-free regions (PERMANOVA test; *F*_*1,27*_ = 2.41, R^2^ = 0.06, *p* = 0.02), while at higher depths (meso- and bathypelagic) there was no apparent difference in community composition between the two regions (PERMANOVA test; *p* > 0.05). In ice-covered regions the dissimilarity between FL and PA communities strongly increased along the entire water column (Fig. 3b; Wilcoxon Signed-Ranks Test; *p*-adjusted *p* < 0.001), in contrast to ice-free region where the dissimilarity between the fractions increased mainly between the epi- and mesopelagic depths (Fig. 3b; Wilcoxon Signed-Ranks Test; *p*-adjusted *p* < 0.001). Thus, this suggests a higher exchange between communities of the FL and PA fraction (i.e., via particle colonization) in the ice-free region.

### Vertical connectivity and shifts in particle-associated communities

**Fig. 4:**
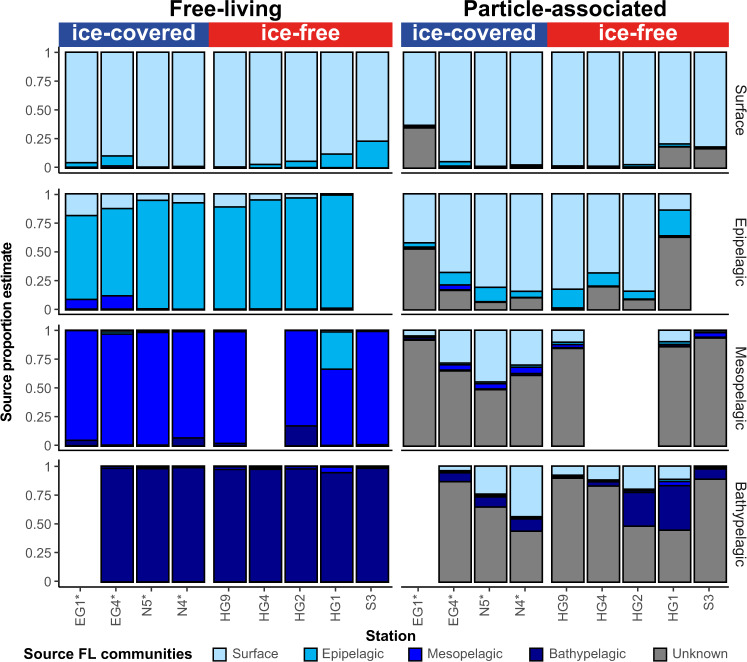
Proportion estimates of the source water masses for microbial communities in different water layers. Each bar plot represents the fraction of the microbial community linked to its most probable water mass source, estimated using ‘SourceTracker’. The source estimates for each free-living (FL) community was conducted estimated using leave-one-out approach (i.e., based on all other FL communities; see methods), and the sources of the particle-associated (PA) communities were estimated based on the FL communities. The ice-covered stations are marked with an asterisk.

Many free-living (FL) microbes are adapted to colonize particles in the water column. Thus, the observed vertical dissimilarity pattern of the PA communities could be associated with colonization by different FL microbes, and due to the diversity changes in the FL communities as a function of water depth we can test aggregate colonization by FL microbes through the water column. In order to test where and which FL communities colonized the settling aggregates, we applied a microbial source tracking (MST) Bayesian algorithm ‘SourceTracker’. This MST approach assumes that ASVs diversity in various ‘source’ (i.e., FL) and corresponding ‘sink’ (i.e., PA) communities allows identification of statistically probable links between them (for detailed explanation see Methods section). The MST analysis showed a strong link between the surface and epipelagic FL microbes and the composition of PA communities throughout the entire water column (Fig. 4). Within the surface and epipelagic layers, a particularly high proportion (84 ± 5%) of the PA communities was estimated to originate from surface and epipelagic FL communities. In contrast, at meso- and bathypelagic depths the PA communities showed that only a small proportion of the community originated from the meso- and bathypelagic FL communities (ca. 2 and 8% of the communities, respectively), and potential source of a large fraction of the communities (72 ± 5%) was not identified by the algorithm. However, at meso- and bathypelagic depths, 27 ± 6% of the PA communities in ice-covered and 11 ± 2% of PA communities in ice-free regions were identified to originate from surface and epipelagic FL communities (Fig. 4; Supplementary Data 3).

**Fig. 5:**
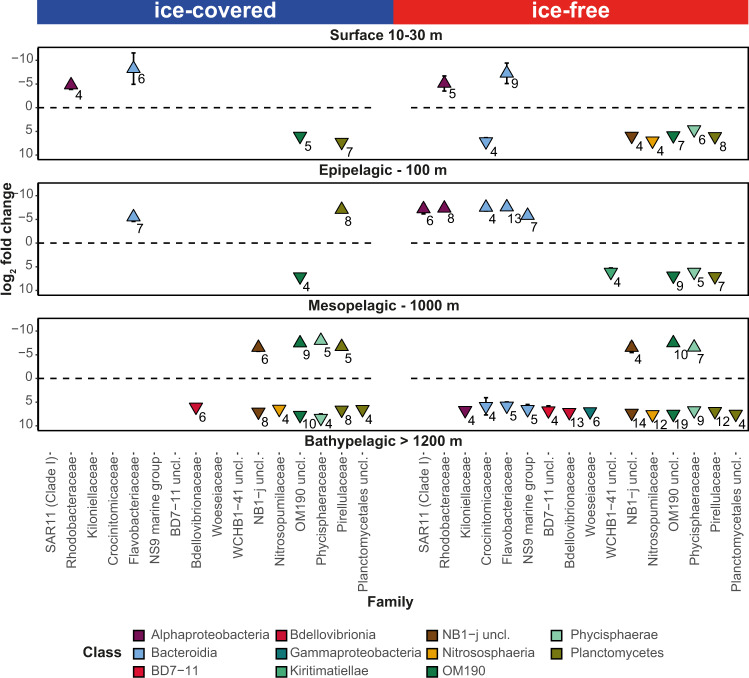
Differences in particle-associated (PA) community composition between the distinct water layers, in ice-covered and ice-free regions. Enriched taxonomic families between each two consecutive depths (surface-epipelagic, epipelagic-mesopelagic and mesopelagic-bathypelagic), ordered according to labels between the panels. The *y*-axis represents the mean log_2_ fold-change for microbial families with more than 3 ASVs with log_2_ fold-change absolute value higher than 1 (standard error is smaller than the point). Positive value represent enrichment in deeper water layers and negative value represents enrichment in shallower water layer. The numbers near the symbols represent the number of ASVs enriched in the depth. The *x*-axis is ordered according to the different taxonomic classes, represented by the color code.

By statistical tests of comparative sequence enrichment, we identified the microbial taxonomic groups that became significantly more abundant on sinking particles as a function increasing depth. The ASVs within the PA communities were defined as enriched when they had a log_2_ fold-change of absolute value higher than 1 (i.e., double the amount of sequences) and an adjusted *p* value lower than 0.1 (Fig. 5). This test looked at consecutive pelagic layers: surface-epipelagic, epipelagic-mesopelagic and mesopelagic-bathypelagic. In both ice-free and ice-covered regions, PA communities became enriched with increasing depth in the classes *Gammaproteobacteria* (with 40 and 18 enriched ASVs, respectively), *Planctomycetes* (with 37 and 27 enriched ASVs, respectively), *Bacteroidia* (43 and 8 enriched ASVs, respectively), and the poorly characterized class OM190 (with 34 and 19 enriched ASVs, respectively; Supplementary Data 4). The enriched ASVs of these classes reached up to 5% of the sequences in the PA communities of the ice-covered regions and up to 10% of the sequences in the ice-free region. However, while the enriched ASVs of the classes *Gammaproteobacteria* and *Bacteroidia* were also present in the FL communities, the enriched ASVs of the classes *Planctomycetes* and OM190 were absent from the FL fraction (<0.5% of the sequences). Overall, we observed larger changes with increasing depth in the PA communities from the ice-free region (where sinking velocity of particles was lower), resulting in more than double the amount of PA-enriched ASVs, in comparison to the ice-covered regions (348 and 158 ASVs, respectively).

### Transport of surface water-originating microbes to the bathypelagic: water column vs. seafloor

**Fig. 6:**
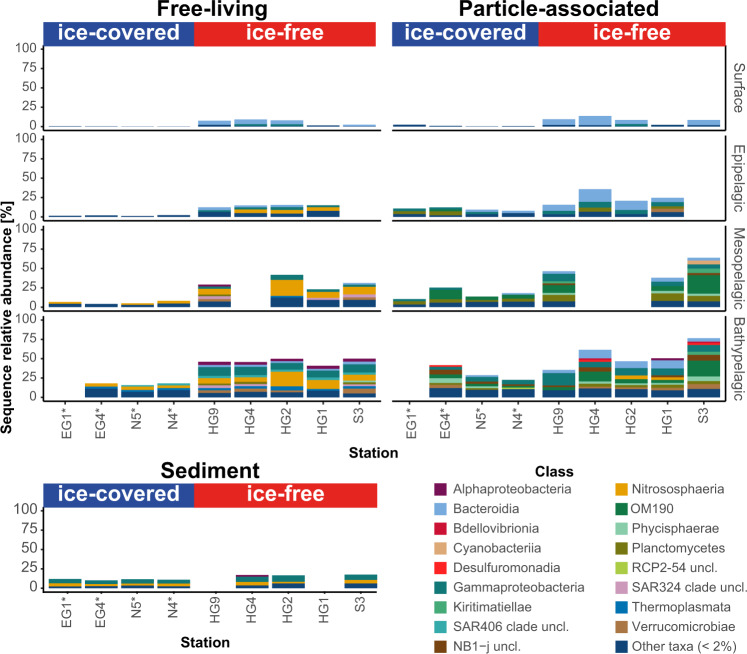
Relative sequence abundance of PA-enriched ASVs in water column and sediment microbial communities. Only the sequence proportion of ASVs that were found enriched along the water column in PA communities were included in the figure. The classes represented by colors according to the legend, all classes with sequence proportion below 2% were classified as “Other taxa”. The ice-covered stations are marked with an asterisk.

Some of the vertically PA-enriched ASVs were also present in the FL communities along the water column (Fig. 6). In the bathypelagic, the PA-enriched ASVs comprised 17 ± 2% of the sequences in the FL communities from the ice-covered region, and 47 ± 4% of the sequences in the FL communities from the ice-free region. The most abundant family that consisted of such ASVs was the archaeal family *Nitrosopumilacea*, which comprised 3–4% and 6–19% of sequences in FL communities of the ice-covered and ice-free regions, respectively.

The seeding of the deep-sea sediment by microbes on sinking particles was tested using seven deep-sea sediment microbial communities (uppermost centimeter) that were sampled at the same stations as the water column across the Fram Strait. These communities were characterized using a similar approach to the FL and PA communities in the water column, and the final dataset consisted of 1,209,785 sequences that were assigned to 11,145 ASVs associated with bacterial and archaeal lineages (Supplementary Data 2; Supplementary Fig. 3). The sediment microbial communities were mainly affiliated to the classes *Alphaproteobacteria*, *Gammaproteobacteria*, and *Nitrososphaeria* (Supplementary Fig. 4).

The vertically PA-enriched ASVs were also identified in the deep-sea sediment communities of both ice-covered and ice-free regions (Fig. 6). These shared ASVs between the PA and the sediment communities were mainly associated with the archaeal family *Nitrosopumilaceae* (17 ASVs) and the bacterial family *Woeseiaceae* (8 ASVs; class *Gammaproteobacteria*), each family comprised ca. 2–4% of the sequences in the sediment communities. Interestingly, in contrast to the PA-enriched ASVs of the family *Nitrosopumilaceae* that were also abundant in the FL communities of the bathypelagic, the shared ASVs of the family *Woeseiaceae* were absent from the FL communities (<0.3% of sequences in all FL communities). Overall, in ice-free region, 109 of the PA-enriched ASVs were present in the sediment comprised and comprised ca. 17% of the sequences in them. In contrast, in ice-covered regions, 61 of the PA-enriched ASVs were present in the sediment and comprised ca. 11% of the sequences in them (Fig. 6).

## Discussion

Throughout the world’s oceans, settling particles export organic matter and nutrients, as well as microbes and their enzymes, to the deep ocean^20,22,23,27^. However, little is known about the influence of Arctic sea ice dynamics on the composition and sinking velocities of settling particles and hence on export flux and export efficiency in Arctic waters. This is due to difficulties with the exchange of sediment traps and appropriate year-round upper ocean observations in ice-covered regions, which limit microbiological and biogeochemical deep-sea studies in the Arctic Ocean^35,28^. From what is known, snow and ice cover affect productivity by light limitation, and thereby carbon export is relatively low under the sea ice^31,36^. However, recently, the ice cover thinned substantially, so that it has lesser effect on light limitation and rafting of particles in the Central Arctic basins and in the Fram Strait^29,30^. Furthermore, it was found that the sea ice margin can have stimulating effects on primary production early in the season, by meltwater-induced stratification and through seeding with ice-associated primary producers^18,37^. Spatially and temporarily this can lead to higher export efficiencies and stronger pelagic-benthic coupling in regions with seasonal presence of the sea ice margin, or covered partially by thinning sea ice, including in the Fram Strait^38^, in the regions north of Svalbard^39^, as well as in the Central Arctic^16^.

Here, we studied the role of sea ice on settling particle characteristics and vertical microbial connectivity, and postulate links to carbon export efficiency in the Fram Strait. Our long-term assessment of the role of ice-coverage on particle export during periods with sea ice near the HG4 station suggested an important function of sea ice distance on export fluxes early in spring during the ice-influenced phytoplankton bloom period. On the other hand, during the summer (June–August), when the sea ice was much further from the trap, there was no clear relationship between the export flux and the distance to the ice-edge. This encouraged us to assess the underlying principles of this connection between ice-associated export and the fate of microbial communities attached to particles close to the sea ice edge. To test how ice-coverage impacts vertical connectivity and export of organic matter, we compared characteristics of sinking particles from ice-covered and ice-free regions of the Fram Strait during the productive season. At the time of sampling in June–July 2016, the phytoplankton bloom was dominated by diatoms in the ice-covered region while in the adjacent ice-free region it was dominated by *Phaeocystis* spp. In the ice-covered region we found larger diatom aggregates, with two-fold higher size-specific sinking velocities compared to the smaller *Phaeocystis* spp. aggregates that dominated ice-free regions. This caused two-fold higher carbon export rates under the sea ice, compared to adjacent ice-free waters during the same period. The long-term record in the Fram Strait also shows that annual particle flux is lower during warm water phases with less ice^40^, and characterized by a shift from diatom to coccolithophorid and *Phaeocystis* spp. dominated phytoplankton during summertime at HG4 station^41^. This is similar to observations north of Svalbard where ice-associated diatom production resulted in higher export than that observed for ice-free regions dominated by *Phaeocystis* spp^39^. Taken together, this suggests that in the early Arctic summer, fast-settling diatom aggregates drive export in ice-covered regions, whereas in warming, Atlantic-water influenced regions, the slower settling *Phaeocystis* spp. aggregates dominate and lead to more pelagic recycling. In our study, this also affected carbon to nitrogen ratios of the sinking matter, which were lower for the settling particles collected by the drifting traps in the ice-covered regions compared to the ice-free region, suggesting that ‘fresher’ organic material was exported by the fast-settling, ice-associated diatom aggregates. It was recently shown that phytoplankton growth may occur in early spring under the sea ice^42^, it is therefore possible that the higher ice-associated POC flux was caused by higher production in the ice-covered region. However, we did not observe any differences in nutrient and phytoplankton concentrations between the ice-covered (‘N’) and the ice-free (‘HG’) regions, suggesting that it was the differences in aggregate settling and size, rather than differences in production that caused the increased POC export in the ice-free region.

There are of course large uncertainties associated with our method of using contrasting ice conditions during summer to study the mechanisms that may explain the relationship between POC flux and seasonal ice coverage. For example, thinning of snow cover and ice thickness will promote growth of sympagic and pelagic algae, while complete ice loss will remove sympagic algae but also allow earlier growth of pelagic phytoplankton^43,44^. However, the POC export from the short-term drifting traps deployed at 100 m during June–July in 2016 matched well with the historical POC flux collected at 200 m between 2001 and 2013, when using the common method to estimate POC flux attenuation between 100 and 200 m via a power law function^45^ (see Supplementary Discussion). Hence, a potential future shift due to Atlantification of the Eurasian Arctic Basin^46^, with larger areas of thermally stratified open waters, a loss of sympagic production, flagellate-dominated phytoplankton blooms, slower settling aggregates and stronger grazing pressure, may lead to higher degradation and transformation of organic matter during its journey through the water column. This will lead to lower amounts and less labile organic matter reaching the seafloor.

In order to estimate the “fate” of sinking particles once they sink out of the ice-covered and ice-free surface waters towards the deep ocean, we modeled their sinking trajectories by combining a physical oceanographic model^47^ with the observed on-board particle sinking velocities. The implemented ocean-sea ice model is capable of resolving the complex mesoscale eddy dynamics of the Fram Strait and has been shown to realistically simulate its ocean dynamics^48^. The Lagrangian trajectories were simulated using constant sinking velocity throughout the water column, based on measurements conducted on particles collected in the epipelagic waters (60 m). Yet, in the ocean, during the sinking particles may change their velocities, due to microbial degradation and fragmentation by zooplankton^49,50^. To investigate the impact that this might have on our results, we expanded the trajectory analysis with two scenarios to represent the realistic extremities of fast- and slow-sinking phytoplankton aggregates in the upper 1000 m of the water column in the region (i.e., average slow-sinking aggregates with 20 m d^−1^ and average fast-sinking aggregates with 60 m d^−1^). The trajectories of the slow- and fast-sinking aggregates observed at 1000 m depth were then determined for both the ice-free and ice-covered regions. In all cases, the simulated trajectories revealed that during sinking to the deep ocean only a small fraction of the particles were horizontally moved from ice-covered to ice-free regions, and vice versa. This suggested that the observed differences in vertical microbial connectivity from the surface to the deep ocean were not caused by horizontal movement of the aggregates between ice-free and ice-covered regions.

In this study, we tested the previously established hypothesis that vertical microbial connectivity is stronger in ecosystems dominated by fast-settling aggregates^22,23,25^, due to the shorter transit time through the water column. In both ice-covered and ice-free regions of the Fram Strait, free-living (FL) pelagic microbial communities from different depths had greater dissimilarities to each other than the particle-associated (PA) communities from the same depths. This suggests a stratified water column with distinct microbial communities in the different water layers, as well as a vertical dispersal of microbial communities between surface and deep ocean via sinking particles. In this context, settling particles are not only important for the export of organic matter to the deep ocean, but they also promote microbial heterotrophic activity and seeding^22,23,25,26,28^, and thereby shape microbial biogeography and biogeochemical functioning in meso- and bathypelagic realms.

The surface water-originating microbial families that were significantly enriched on particles collected at depth, such as various members of the class *Bacteroidia*, are associated with phytoplankton blooms in the region^35,51^, and are known to be highly active organic matter degraders^52^. Furthermore, it has recently been shown that there is a dominance of enzymatic activity phylogenetically linked to these taxonomic groups in the bathypelagic^53^ and that this enzymatic activity is predominantly linked to a particle-associated lifestyles^54^. This indicates that active microbes originating from surface waters and associated with sinking particles continue to process organic matter while they sink to the deep ocean, and thus may remain key players in the biogeochemical cycling in the deep ocean. The cold water-adapted (i.e., psychrophilic) microbes of polar waters may potentially thrive at depth of the Arctic Ocean, which is characterized by a relatively uniform temperature. Our results show that almost half of the bathypelagic FL communities consisted of vertically PA-enriched ASVs. This suggests that surface water-originating microbes may realize an ecological niche in bathypelagic waters. Evidence for this is provided by the archaeal family *Nitrosopumilaceae*, which was the most abundant among taxonomic groups with PA-enriched ASVs in bathypelagic FL communities. Previous analyses showed that epipelagic and bathypelagic members of this family are phylogenetically closer to each other than those found in the intermediate mesopelagic waters^55^. Based on our results, this pattern may be explained by a niche realization in the bathypelagic of *Nitrosopumilaceae* family members exported from the surface ocean on sinking particles.

In both ice-free and adjacent ice-covered regions, the high similarity between surface PA and FL communities suggested that particles were colonized in surface waters, similar to other oceanic regions^22,23,25–27^. Even in the bathypelagic, a substantial proportion of the PA community was still comprised of microbes recruited in the surface ocean. Notably, we observed an increasing dissimilarity between FL and PA communities with depth, and particles in the meso- and bathypelagic contained a high proportion of sequences that were not linked to the FL community at any depth, indicating a shift in population densities in the particle, e.g., by growth of otherwise rare microbial clades in the particles. Alternatively, the relatively long sinking time of days to weeks could have led to PA microbial communities at depth containing an imprint of surface water’s microbial communities that were no longer present or horizontally offset during our sampling. One of the known weaknesses of the applied here MST method is its limited ability to differentiate between sources with very similar communities^56^. Thus, a fraction of the unidentified sources of PA communities at depth could be a result of internal constrains of the predictive algorithm. We further observed higher dissimilarity between FL and PA communities in surface and epipelagic waters of the ice-covered region where fast-sinking particles were more abundant, compared to the ice-free region. Overall, we conclude from our data that the observed vertical changes in the PA communities are substantially affected by sinking velocity, causing different encounter rates and colonization in the surface ocean^57^, and differences in time for ecological succession within the particles^58^. The succession can result from transformations in the aggregate composition during aging and turnover^59^, grazing by protozoans^60^, viral infection^61^, or changing environmental conditions, such as increasing hydrostatic pressure^62,63^. Common to all these processes is that on slower sinking particles there will be more time for a stronger selection of some taxonomic groups and the demise of others, potentially allowing rare taxa to become abundant at depth while those that were abundant at shallower depths become rarer. Furthermore, a large fraction of sinking particles remains suspended in the bathypelagic^64^. In this way, settling aggregates should be viewed as constantly changing microcosms that have some exchange with their surroundings in the deep ocean^27^, but where particle sinking velocity is an important driver of succession. In Arctic waters, it seems that fast aggregate sinking velocity is strongly related to ecological impact from sea ice cover.

Since the seafloor is the final destination for those particles that make the journey through the water column, we tested whether the vertical microbial connectivity extends to deep-sea sediment. We found that ca. 10–20% of the sequences in the deep-sea sediment were related to PA-enriched microbes originating from the surface waters and deposited via sinking particles, in both ice-covered and ice-free regions. These results are comparable to observations in the Central Arctic Ocean after deposition of ice algae on the deep seafloor^28^, and are higher than the global average of <10%^65^. Interestingly, the family *Woeseiaceae* (class *Gammaproteobacteria*) showed the strongest benthic-pelagic connectivity via sinking particles, indicating its export from surface to bathypelagic waters via sinking particles^22^. Recent genomic characterization of this largely unknown taxonomic group (which was conducted using sediment samples collected in the Fram Strait) suggests their involvement in the cycling of detrital proteins in marine benthic environments^66^. Using targeted fluorescence microscopy of the total pelagic microbial communities (based on samples collected in parallel to this study), Hoffmann et al.^66^ also showed, that cells of this taxonomic group were present throughout the entire water column, comprising < 1% of the community. We found that pelagic members of the *Woeseiaceae* were associated with sinking particles, but not free-living, suggesting that this important benthic heterotroph is one of the few types of bacteria that cover all water depths by a particle-associated life style.

In conclusion, our study supports the notion that sea-ice retreat can have an important ecological impact on carbon flux characteristics, and on long-term potentially affect the deep-ocean microbial diversity. Fast-settling ice-associated diatom aggregates drive higher export efficiency and cause stronger pelagic-benthic coupling including the transport of functionally important microbial groups, whereas slow settling *Phaeocystis* spp. aggregates associated with seasonally ice-free regions may lead to more pelagic recycling and less vertical connectivity. These changes may substantially alter deep water and seafloor communities in the Arctic Ocean.

## Methods

### Water sampling and metadata collection

The sampling was performed on board of RV Polarstern during expedition PS99.2 to the LTER observatory HAUSGARTEN in the Fram Strait (June 24–July 16, 2016). Water samples were collected using 12 L Niskin bottles mounted on a CTD rosette (Sea-Bird Electronics Inc. SBE 911 plus probe) equipped with double temperature and conductivity sensors, a pressure sensor, chlorophyll *a* fluorometer, and transmissometer. At all stations water samples were collected from surface at 10–30 m, 100 m, 1000 m and ~50 m above the seafloor (Supplementary Data 2). For assessing archaeal and bacterial community composition, 4 L in epipelagic (<100 m) and 8–12 L in meso- and bathypelagic waters were collected from the Niskin bottles into clean jerry cans, and were filtered with a peristaltic pump (Masterflex; Cole Parmer) through successive membrane filters of 5 µm (Whatman Nucleopore, 47 mm polycarbonate), and 0.22 µm (Millipore Sterivex™ filters). The filtration process took place for up to 2 h and was conducted at 4 °C. In addition, deep-sea sediment cores were collected with a TV-guided multicorer, and subsamples of the uppermost centimeter of the cores were collected with syringes (Supplementary Data 2). All samples were stored at −20 °C until DNA isolation.

Hydrographic data of the seawater including temperature and salinity (doi:10.1594/PANGAEA.871952), as well as the inorganic nutrient concentrations (doi:10.1594/PANGAEA.906132) were retrieved from PANGAEA. Inorganic nutrient consumption (Δ) at each station was calculated by subtracting the mean value of all collected measurements above 50 m from the mean value of all collected measurements between 50 and 100 m (below the seasonal pycnocline)^51^.

The map in Fig. 1a was generated using ArcMap (v10.5) with Esri world countries dataset (www.esri.com) in a WGS 1984 Arctic Polar Stereographic map projection. The mean monthly sea ice concentrations for Fig. 1a were retrieved from http://data.seaiceportal.de^67^, and sea surface temperature was obtained from NOAA NCEP real-time analysis (http://polar.ncep.noaa.gov/sst/rtg_high_res/).

### Long-term sediment trap deployment and sea-ice distance estimation

The long-term moored KIEL sediment trap (sampling area 0.5 m^2^ and 20 collection cups) was deployed and recovered yearly from 2001 to 2013 at the central LTER observatory HAUSGARTEN station (HG4 − 79.01°N, 4.20°E; Fig. 1). The deployment depths of the sediment trap was ~ 200 m. Sampling cups were filled with filtered seawater adjusted to a salinity of 40 and fixed with 0.14% final solution of HgCl_2_. The opening time of the sampling cups varied between 7 and 59 days, depending on the season with short opening time during the polar day and long opening time during the polar night. In the laboratory, swimmers (e.g., *Amphipoda*, *Copepoda*, *Pteropoda*, *Chaetognatha*) were manually removed with forceps and rinsed under a dissecting microscope. Triplicate subsamples were measured for particulate organic carbon (POC) by filtering the material onto pre-combusted Whatman GF/F filters, soaking them in 0.1 N HCl, and drying at 60 °C before analyzing with a CHN elemental analyzer^14,38^. The data was retrieved from PANGAEA (doi: 10.1594/PANGAEA.855473).

To evaluate the impact of the sea ice concentration on the POC flux during periods when peak POC flux was associated with sea ice (March-May), we used POC flux collected by the long-term sediment trap of the central station HG4. The daily distance between the sea-ice edge and HG4 was estimated using daily sea ice concentration satellite images from NSIDC/NOAA (http://nsidc.org/data/nsidc-0051). The images were generated using the NASA Team algorithm^68^ and mapped to a 25 × 25 km grid. This satellite dataset was derived from brightness and temperature data generated from Scanning Multichannel Microwave Radiometer and Sensor Microwave Imager and Sounder equipped on the Nimbus-7 satellite and the Defence Meteorological Satellite Program, respectively. The distance to the ice edge was defined at the position with 15% sea ice concentration. The ice-edge nearest the HG4 position was used to calculate the daily ice distance and averaged for each opening time of the collection cups (~14 days) on the long-term moored sediment traps.

### Modeled aggregates sinking trajectories

A Lagrangian particle tracking algorithm was used to backtrack particles from the sampling depth to the surface. A detailed description of the model can be found in Wekerle et al.^32^. Briefly, the backward particle computation is done by reversing the flow field, i.e. particles are treated as if they were rising from the sampling depth to the surface with a negative sinking velocity, being horizontally displaced with the reversed horizontal velocity. Particles were advected with daily averaged 3D model velocities from the ocean general circulation model FESOM (an ocean-sea ice model based on unstructured meshes)^47^. The particle sinking velocity was computed by adding a constant sinking velocity to the modeled vertical velocity. In this study, we used a FESOM configuration optimized for the Fram Strait, applying a mesh resolution of 1 km^48^. The performance of the model was validated for the sampling time period by oceanographic observations (Supplementary Fig. 6).

Particles were “released” around 300 m above the seafloor once per day during the year 2016, however we restricted the analysis to particles that reached the ocean surface between March and July 2016 (i.e., during the productive season). A time step of 30 min was used for the trajectory calculation, and bi-hourly positions and corresponding temperature and salinity values were stored. To quantify the vertical distribution of particles, their positions were binned into a grid with bin sizes of 25 m depth × 0.05° Longitude/Latitude and then divided by the total number of particles to determine the fraction of particles originating from each grid box. The daily concentrations of sea ice were retrieved from Centre d’Exploitation et de Recherche SATellitaire (CERSAT; http://cersat.ifremer.fr/).

### Microscopic analysis of phyto- and protozooplankton

The plankton community composition at the chlorophyll *a* maximum was identified and the phytoplankton abundance was counted using light microscopy. Seawater samples were preserved in hexamethylenetetramine-buffered formalin (final concentration 0.5–1%) and stored in brown glass bottles. For microscopic analyses an aliquot of 50 mL was transferred to Utermöhl settling chambers where the cells were allowed to settle for 48 h. At least 500 cells of the dominant phytoplankton species or groups were counted with an inverted microscope at three different magnifications using phase contrast according to Edler et al.^69^.

### On-board characterization of marine aggregates and sinking velocity measurements

Using a marine snow catcher (MSC, OSIL, United Kingdom) we sampled intact aggregates from 60 m at ice-free and ice-covered regions, and measured their size, composition, and sinking velocities. The aggregates were individually transferred to a vertical flow chamber^70^ filled with Whatman GF/F filtered seawater collected from the same MSC and kept at in situ temperature. The *x*-, *y*-, and *z*-axes of each aggregate were measured in the vertical flow system using a horizontal dissection microscope and an ocular with a scale. The aggregate volume was thereafter calculated assuming an ellipsoidal shape and the equivalent spherical diameter (ESD) was calculated from the aggregate’s volume. The sinking velocity was measured by increasing the upward flow in the flow-chamber until the aggregate was floating one diameter above the net. The sinking velocity was thereafter calculated by determining the volumetric flow rate three times, and dividing the average of these measurements by the area of the flow chamber. The composition of the aggregates was determined with an inverted light microscope using Utermöhl chambers (Fig. 2).

### Aggregate and carbon export to 100 m

Aggregate and carbon export to 100 m depth was measured using the free-drifting surface-tethered sediment traps in the ice-free and ice-covered regions^27^. The drifting traps consisted of a drifting array attached to a surface buoy equipped with a GPS satellite transmitter, two surface floats and 12 small buoyancy balls that served as wave breakers to reduce hydrodynamic mixing effects on the sediment traps. The 100 m collection depth was equipped with four gimbal-mounted cylinders, each 1 m tall and 10.4 cm in inner diameter. Three of the cylinders collected samples for biogeochemical measurements and the last collection cylinder contained 200 ml of a viscous gel, which intercepted and preserved settling particles without destroying their original sizes and structures. Upon recovery, the material collected for biogeochemical fluxes was fixed with HgCl_2_ and stored at 4 °C until further analyses in the home laboratory. The particles collected in the gels were photographed using a stereo microscope equipped with a 3.1 megapixel digital camera and a 105 mm macro lens, resulting in a pixel size of 12 µm. The image analyses were performed with a routine written in MATLAB (The MathWorks) using the image analysis toolbox. Each image was converted into gray scale and the background was removed by applying a threshold value. The calibrated pixel area (mm^2^) in each projected particle was converted into the equivalent spherical diameter (ESD).

### DNA isolation and 16S rRNA amplicon analysis

Genomic bacterial and archaeal DNA was isolated from size-fractionated filtration through 5 µm and 0.22 µm filters membranes to analyze the particle-associated (PA, > 5 µm) and the free-living (FL, > 0.22 µm and <5 µm) communities. The isolations were conducted by a combined chemical and mechanical procedure using the PowerWater DNA Isolation Kit, and PowerSoil DNA Isolation Kit for the sediment samples (MO BIO Laboratories, Inc., Carlsbad, CA, USA). Prior to DNA isolation the Sterivex™ cartridges of the 0.22 μm membranes were opened in order to place the filters in the kit-supplied bead beating tubes. The isolation was continued according to the manufacturer’s instructions, and the DNA was stored at −20 °C. Library preparation was performed according to the standard instructions of the 16S Metagenomic Sequencing Library Preparation protocol (Illumina, Inc., San Diego, CA, USA). The hyper variable V4–V5 region of the 16S rRNA gene was amplified using bacterial primers 515F-Y (5‘-GTGYCAGCMGCCGCGGTAA-3‘) and 926 R (5‘-CCGYCAATTYMTTTRAGTTT-3‘)^71,72^. Sequences were obtained on the Illumina MiSeq platform in a 2 × 300 bp paired-end run (CeBiTec Bielefeld, Germany), following the standard instructions of the 16S Metagenomic Sequencing Library Preparation protocol (Illumina, Inc., San Diego, CA, USA).

The raw paired-end reads were primer-trimmed using cutadapt (v3.0)^73^. Further analyses were conducted using R (v3.6.3; http://www.Rproject.org/) in RStudio (v1.2.5033; http://www.rstudio.com/). The trimmed libraries were processed using DADA2 (v1.14.1)^33^, following the suggested tutorial (https://benjjneb.github.io/dada2/tutorial.html). Briefly, chimeras and singletons were filtered out. The produced amplicon sequence variants (ASVs) were taxonomically classified against the SILVA 16S rRNA gene reference database (release 138)^74^. The ASVs that were taxonomically unclassified on domain level, or not assigned to bacterial or archaeal lineages, were excluded from further analysis. Furthermore, all ASVs which were taxonomically assigned to mitochondria and chloroplast were removed from the dataset. Overall, ca. 25% of the total raw sequences were filtered out from the final dataset.

### Statistics and reproducibility

Sample data matrices were managed using the R package ‘phyloseq’ (v1.28.0)^75^ and plots were generated using R package ‘ggplot2’ (v3.3.0)^76^. The sample rarefaction analyses were conducted using R package ‘iNEXT’ (v2.0.20)^34^. Prior to beta-diversity analyses, a prevalence threshold (i.e., in how many samples did an ASV appeared at least once) of 4% was applied on the ASV abundance table. Principal component analysis (PCA) and dissimilarity comparisons between FL and PA communities were conducted on a variance stabilized ASV abundance table based on the geometric mean^77^. The fold-change in abundance of each ASV between the water layers was calculated using the R package ‘DEseq2’ (v1.24.0)^78^. The method applies a generalized exact binomial test on variance stabilized ASV abundance.

Based on the assumption that the particle-associated microbial communities (i.e., ‘sink’ communities) are the result of various events of colonization of marine aggregates by free-living microbes (i.e., ‘source’ communities); a Bayesian community-based microbial source tracking algorithm ‘SourceTracker‘ (v1.0)^79^ was applied on the ASV abundance table. The SourceTracker approach models the potential presence of a mixture of the source communities in the sink communities, where the mixing proportions of the different sources are unknown. The algorithm performance was validated using a ‘leave-one-out’ approach, in which each ‘source’ (i.e., FL) community was hidden, in turn, from the training dataset, and its origin was predicted based on the rest of the source samples in the dataset. All samples were randomly sub-sampled to 5,000 sequences prior to the SourceTracker analysis. The SourceTracker analysis was conducted according to default conditions, defined by the developers: burn-in period—100, restarts—10, dirichlet hyperparameters (ɑ, β)—0.001 (the algorithm and its configuration are described in detail in ref. ^79^).

### Reporting summary

Further information on research design is available in the Nature Research Reporting Summary linked to this article.

## Supplementary information

Supplemental Material

Description of Additional Supplementary Files

Supplementary Data 1

Supplementary Data 2

Supplementary Data 3

Supplementary Data 4

Supplementary Data 5

Reporting Summary

## Data Availability

Raw paired-end, primer-trimmed reads were deposited in the European Nucleotide Archive (ENA)^80^ under accession number PRJEB30254. The data were archived using the brokerage service of the German Federation for Biological Data (GFBio)^81^. The modeled backward particle trajectories were deposited on PANGAEA data publisher under^82^.

## Code availability

Scripts for the molecular data processing and statistical analyses can be accessed via Zenodo under^83^.

## References

[1] Serreze MC, Meier WN (2019). The Arctic’s sea ice cover: trends, variability, predictability, and comparisons to the Antarctic. Ann. N. Y. Acad. Sci..

[2] Pörtner, H. et al. IPCC, 2019: IPCC Special Report on the Ocean and Cryosphere in a Changing Climate. *Intergov. Panel Clim. Chang*. 1–765 (2019).

[3] Kwok R (2018). Arctic sea ice thickness, volume, and multiyear ice coverage: losses and coupled variability (1958–2018). Environ. Res. Lett..

[4] Wassmann P, Reigstad M (2011). Future Arctic Ocean seasonal ice zones and implications for pelagic-benthic coupling. Oceanography.

[5] Nöthig E-M (2015). Summertime plankton ecology in Fram Strait—a compilation of long- and short-term observations. Polar Res..

[6] Assmy P (2017). Leads in Arctic pack ice enable early phytoplankton blooms below snow-covered sea ice. Sci. Rep..

[7] Neukermans G, Oziel L, Babin M (2018). Increased intrusion of warming Atlantic water leads to rapid expansion of temperate phytoplankton in the Arctic. Glob. Chang. Biol..

[8] Wiedmann, I. et al. What feeds the Benthos in the Arctic Basins? Assembling a carbon budget for the deep Arctic Ocean. *Front. Mar. Sci*. **7**, 544386 (2020).

[9] Randelhoff A, Sundfjord A (2018). Short commentary on marine productivity at Arctic shelf breaks: upwelling, advection and vertical mixing. Ocean Sci..

[10] Lewis KM, van Dijken GL, Arrigo KR (2020). Changes in phytoplankton concentration now drive increased Arctic Ocean primary production. Sci. (80-.)..

[11] Arrigo KR, van Dijken GL (2015). Continued increases in Arctic Ocean primary production. Prog. Oceanogr..

[12] Leu E (2015). Arctic spring awakening—steering principles behind the phenology of vernal ice algal blooms. Prog. Oceanogr..

[13] Arrigo KR (2012). Massive phytoplankton blooms under Arctic sea ice. Science.

[14] Lalande C (2014). Variability in under-ice export fluxes of biogenic matter in the Arctic Ocean. Glob. Biogeochem. Cycles.

[15] Fernández-Méndez M (2015). Photosynthetic production in the central Arctic Ocean during the record sea-ice minimum in 2012. Biogeosciences.

[16] Boetius A (2013). Export of algal biomass from the melting Arctic sea ice. Science.

[17] Assmy P (2013). Floating ice-algal aggregates below melting Arctic sea ice. PLoS ONE.

[18] Perrette M, Yool A, Quartly GD, Popova EE (2011). Near-ubiquity of ice-edge blooms in the Arctic. Biogeosciences.

[19] Underwood GJC (2019). Organic matter from Arctic sea-ice loss alters bacterial community structure and function. Nat. Clim. Chang..

[20] Herndl GJ, Reinthaler T (2013). Microbial control of the dark end of the biological pump. Nat. Geosci..

[21] Henson S, Le Moigne F, Giering S (2019). Drivers of carbon export efficiency in the global ocean. Glob. Biogeochem. Cycles.

[22] Ruiz‐González C (2020). Major imprint of surface plankton on deep ocean prokaryotic structure and activity. Mol. Ecol..

[23] Mestre M (2018). Sinking particles promote vertical connectivity in the ocean microbiome. Proc. Natl Acad. Sci. USA.

[24] Preston CM, Durkin CA, Yamahara KM (2020). DNA metabarcoding reveals organisms contributing to particulate matter flux to abyssal depths in the North East Pacific ocean. Deep Sea Res. Part II Top. Stud. Oceanogr..

[25] Poff KE, Leu AO, Eppley JM, Karl DM, DeLong EF (2021). Microbial dynamics of elevated carbon flux in the open ocean’s abyss. Proc. Natl Acad. Sci. USA.

[26] Boeuf D (2019). Biological composition and microbial dynamics of sinking particulate organic matter at abyssal depths in the oligotrophic open ocean. Proc. Natl Acad. Sci. USA.

[27] Thiele S, Fuchs BM, Amann R, Iversen MH (2015). Colonization in the photic zone and subsequent changes during sinking determine bacterial community composition in marine snow. Appl. Environ. Microbiol..

[28] Rapp, J. Z., Fernández-Méndez, M., Bienhold, C. & Boetius, A. Effects of ice-algal aggregate export on the connectivity of bacterial communities in the central Arctic Ocean. *Front. Microbiol*. **9**, 1035 (2018). 10.3389/fmicb.2018.01035 PMC5974969 29875749

[29] Smedsrud LH, Halvorsen MH, Stroeve JC, Zhang R, Kloster K (2017). Fram Strait sea ice export variability and September Arctic sea ice extent over the last 80 years. Cryosphere.

[30] Krumpen T (2019). Arctic warming interrupts the Transpolar Drift and affects long-range transport of sea ice and ice-rafted matter. Sci. Rep..

[31] Lalande C (2016). Lateral supply and downward export of particulate matter from upper waters to the seafloor in the deep eastern Fram Strait. Deep Sea Res. Part I Oceanogr. Res. Pap..

[32] Wekerle, C., Krumpen, T., Dinter, T., Iversen, M. & Salter, I. Origin and properties of sediment trap catchment areas in Fram Strait: results from Lagrangian modelling and remote sensing. *Front. Mar. Sci*. **5**, 4071– 26 (2018).

[33] Callahan BJ (2016). DADA2: High-resolution sample inference from Illumina amplicon data. Nat. Methods.

[34] Hsieh TC, Ma KH, Chao A (2016). iNEXT: an R package for rarefaction and extrapolation of species diversity (Hill numbers). Methods Ecol Evol.

[35] Wilson B (2017). Changes in marine prokaryote composition with season and depth over an Arctic polar year. Front. Mar. Sci..

[36] Leu E, Søreide JE, Hessen DO, Falk-Petersen S, Berge J (2011). Consequences of changing sea-ice cover for primary and secondary producers in the European Arctic shelf seas: Timing, quantity, and quality. Prog. Oceanogr..

[37] Becagli S (2016). Relationships linking primary production, sea ice melting, and biogenic aerosol in the Arctic. Atmos. Environ..

[38] Lalande C, Bauerfeind E, Nöthig E, Beszczynska-Möller A (2013). Impact of a warm anomaly on export fluxes of biogenic matter in the eastern Fram Strait. Prog. Oceanogr..

[39] Olli, K. et al. Food web functions and interactions during spring and summer in the Arctic water inflow region: investigated through inverse modeling. *Front. Mar. Sci*. **6**, 10.3389/fmars.2019.00244 (2019).

[40] Bauerfeind E (2009). Particle sedimentation patterns in the eastern Fram Strait during 2000 – 2005: Results from the Arctic long-term observatory HAUSGARTEN. Deep Sea Res. Part I.

[41] Soltwedel, T. et al. Natural variability or anthropogenically-induced variation? Insights from 15 years of multidisciplinary observations at the arctic marine LTER site HAUSGARTEN. *Ecol. Indic*. 1–14, 10.1016/j.ecolind.2015.10.001 (2015).

[42] Randelhoff A (2020). Arctic mid-winter phytoplankton growth revealed by autonomous profilers. Sci. Adv..

[43] Tedesco L, Vichi M, Scoccimarro E (2019). Sea-ice algal phenology in a warmer Arctic. Sci. Adv..

[44] Lannuzel D (2020). The future of Arctic sea-ice biogeochemistry and ice-associated ecosystems. Nat. Clim. Chang..

[45] Martin JH, Knauer GA, Karl DM, Broenkow WW (1987). VERTEX: carbon cycling in the northeast Pacific. Deep Sea Res. A: Oceanogr. Res. Pap..

[46] Polyakov IV (2017). Greater role for Atlantic inflows on sea-ice loss in the Eurasian Basin of the Arctic. Ocean. Science.

[47] Wang Q (2014). The Finite Element Sea Ice-Ocean Model (FESOM) v.1.4: formulation of an ocean general circulation model. Geosci. Model Dev..

[48] Wekerle C (2017). Eddy-resolving simulation of the Atlantic water circulation in the Fram Strait with focus on the seasonal cycle. J. Geophys. Res. Ocean..

[49] Iversen MH, Ploug H (2013). Temperature effects on carbon-specific respiration rate and sinking velocity of diatom aggregates – potential implications for deep ocean export processes. Biogeosciences.

[50] Briggs N, Dall’Olmo G, Claustre H (2020). Major role of particle fragmentation in regulating biological sequestration of CO 2 by the oceans. Science.

[51] Fadeev, E. et al. Microbial communities in the East and West Fram Strait during sea ice melting season. *Front. Mar. Sci*. **5**, 429 (2018).

[52] Buchan A, LeCleir GR, Gulvik CA, González JM, Gonzalez JM (2014). Master recyclers: features and functions of bacteria associated with phytoplankton blooms. Nat. Rev. Microbiol..

[53] Bergauer K (2018). Organic matter processing by microbial communities throughout the Atlantic water column as revealed by metaproteomics. Proc. Natl Acad. Sci..

[54] Zhao Z, Baltar F, Herndl GJ (2020). Linking extracellular enzymes to phylogeny indicates a predominantly particle-associated lifestyle of deep-sea prokaryotes. Sci. Adv..

[55] Hatzenpichler R (2012). Diversity, physiology, and niche differentiation of ammonia-oxidizing archaea. Appl. Environ. Microbiol..

[56] Brown CM, Mathai PP, Loesekann T, Staley C, Sadowsky MJ (2019). Influence of library composition on sourcetracker predictions for community-based microbial source tracking. Environ. Sci. Technol..

[57] Słomka J, Alcolombri U, Secchi E, Stocker R, Fernandez VI (2020). Encounter rates between bacteria and small sinking particles. N. J. Phys..

[58] Datta MS, Sliwerska E, Gore J, Polz MF, Cordero OX (2016). Microbial interactions lead to rapid micro-scale succesions on model marine particles. Nat. Commun..

[59] Ploug H, Iversen MH, Fischer G (2008). Ballast, sinking velocity, and apparent diffusivity within marine snow and zooplankton fecal pellets: Implications for substrate turnover by attached bacteria. Limnol. Oceanogr..

[60] Kiørboe T, Tang K, Grossart H, Ploug H (2003). Dynamics of microbial communities on marine snow aggregates: colonization, growth, detachment, and grazing mortality of attached bacteria. Appl. Environ. Microbiol.

[61] Proctor LM, Fuhrman JA (1991). Roles of viral infection in organic particle flux. Mar. Ecol. Prog. Ser..

[62] Tamburini C (2009). Effects of hydrostatic pressure on microbial alteration of sinking fecal pellets. Deep Sea Res. Part II: Top. Stud. Oceanogr..

[63] Grossart HP, Gust G (2009). Hydrostatic pressure affects physiology and community structure of marine bacteria during settling to 4000 m: An experimental approach. Mar. Ecol. Prog. Ser..

[64] Bochdansky AB, Clouse MA, Herndl GJ (2016). Dragon kings of the deep sea: marine particles deviate markedly from the common number-size spectrum. Sci. Rep..

[65] Zinger L, Boetius A, Ramette A (2014). Bacterial taxa-area and distance-decay relationships in marine environments. Mol. Ecol..

[66] Hoffmann K (2020). Diversity and metabolism of Woeseiales bacteria, global members of marine sediment communities. ISME J..

[67] Spreen G, Kaleschke L, Heygster G (2008). Sea ice remote sensing using AMSR-E 89-GHz channels. J. Geophys. Res..

[68] Cavalieri, D. J., Parkinson, C. L., Gloersen, P. & Zwally, H. J. Sea Ice Concentrations from Nimbus-7 SMMR and DMSP SSM/I-SSMIS Passive Microwave Data, Version 1. (1996). 10.5067/8GQ8LZQVL0VL

[69] Edler, L. *Recommendations on Methods for Marine Biological Studies in the Baltic Sea. Phytoplankton and Chlorophyll*. (Baltic Marine Biologists BMB, Sweden) (1979).

[70] Ploug H, Jørgensen BB (1999). A net-jet flow system for mass transfer and micro electrode studies in sinking aggregates. Mar. Ecol. Prog. Ser..

[71] Parada AE, Needham DM, Fuhrman JA (2016). Every base matters: assessing small subunit rRNA primers for marine microbiomes with mock communities, time series and global field samples. Environ. Microbiol..

[72] Fadeev E (2021). Comparison of two 16S rRNA Primers (V3–V4 and V4–V5) for studies of Arctic microbial communities. Front. Microbiol..

[73] Martin M (2011). Cutadapt removes adapter sequences from high-throughput sequencing reads. EMBnet. J..

[74] Quast, C. et al. The SILVA ribosomal RNA gene database project: improved data processing and web-based tools. *Nucleic Acids Res.* **41**, 590–596 (2013). 10.1093/nar/gks1219 PMC3531112 23193283

[75] McMurdie PJ, Holmes S (2013). phyloseq: an R package for reproducible interactive analysis and graphics of microbiome census data. PLoS ONE.

[76] Gómez-Rubio, V. ggplot2—elegant graphics for data analysis (2nd edition). *J. Statistical Softw.* **77**, (2017).

[77] McMurdie PJ, Holmes S (2014). Waste not, want not: why rarefying microbiome data is inadmissible. PLoS Comput. Biol..

[78] Love MI, Huber W, Anders S (2014). Moderated estimation of fold change and dispersion for RNA-seq data with DESeq2. Genome Biol..

[79] Knights D (2011). Bayesian community-wide culture-independent microbial source tracking. Nat. Methods.

[80] Silvester N (2018). The European Nucleotide Archive in 2017. Nucleic Acids Res..

[81] Diepenbroek, M. et al. in *Informatik 2014* (eds. Plödereder, E., Grunske, L., Schneider, E. & Ull, D.) 1711–1721 (Gesellschaft für Informatik e.V., 2014).

[82] Wekerle, C. Backward particle trajectories used to estimate the pathways of settling aggregates measured at stations N, HG and EG in Fram Strait. (2021). Available at: 10.1594/PANGAEA.928251.

[83] Fadeev, E. edfadeev/Export_and_vert_conn_FRAM: Published workflow. (2021). Available at: https://zenodo.org/record/5515441.

