# Supplementary material for: Sea ice presence is linked to higher carbon export and vertical microbial connectivity in the Eurasian Arctic Ocean
Source: Commun Biol. 2021 Nov 3;4:1255. doi: 10.1038/s42003-021-02776-w (PMC8566512; doi:10.1038/s42003-021-02776-w)
Supplement: Supplementary file 1 — Supplemental Material [file 42003_2021_2776_MOESM1_ESM.pdf]

Supplementary Information

Sea ice presence is linked to higher carbon export and vertical microbial connectivity in the Eurasian Arctic Ocean

Eduard Fadeev[1,2,#*], Andreas Rogge[1,3], Simon Ramondenc[1], Eva-Maria Nöthig[1], Claudia Wekerle[1], Christina Bienhold[1,2], Ian Salter[1,4], Anya M. Waite[1,5], Laura Hehemann[1], Antje Boetius[1,2,6], Morten H. Iversen[1,6*]

[1]Alfred Wegener Institute, Helmholtz Center for Polar and Marine Research, D-27570 Bremerhaven, Germany
[2] Max Planck Institute for Marine Microbiology, D-28359 Bremen, Germany
[3] Institute for Ecosystem Research, Kiel University, D-24118 Kiel, Germany
[4] Faroe Marine Research Institute, FO 100 Tórshavn, Faroe Islands
[5] Ocean Frontier Institute, NS B3H 4R2 Halifax, Canada
[6] MARUM and University of Bremen, D-28359 Bremen, Germany
[#]Present address: Department of Functional and Evolutionary Ecology, University of Vienna, A-1090 Vienna, Austria

Corresponding Authors:
Eduard Fadeev – eduard.fadeev@univie.ac.at
Morten H. Iversen - morten.iversen@awi.de

Supplementary Discussion

In this study we assume that summer conditions at contrasting stations are representative of the changing export flux as affected by the seasonal cycle of sea ice, i.e. that processes occurring at different locations are representative of different temporal periods. However, the seasonal sea ice is largely affected by the oceanographic dynamics, and the resulting phenology of primary producers is linked to a combination of sea ice features and the available light conditions[1,2]. To test this, we compared the POC fluxes collected at 100 m depth using the short-term sediment traps in the ice-covered and ice-free regions during our research expedition in summer 2016 (Table 2) with the POC flux collected with the moored long-term sediment traps at different seasons at station HG (Fig. 1b and Supplementary Fig. 1). The under-ice export collected at 100 m depth with the short-term sediment trap (station 'N') is larger than the POC flux collected at 200 m using the long-term moored sediment traps during the ice-covered season (March, April and May, Fig. 1). The same was observed when comparing the POC flux collected at 100 m using the short-term sediment trap in the ice-free regions (station 'HG') to the summer POC flux (June, July and August) collected at 200 m using the long-term moored sediment traps. To test if the differences in POC flux between 100 m and 200 m are realistic in terms of the expected attenuation of the POC flux between the two depth, we used a common method to estimate POC flux attenuation which was introduced by Martin et al. (1987)[3]. This method uses a power function to estimate flux attenuation with increasing depth:

$$F_z = F_{zo} \, (z / z_o)^{-b} \qquad (1)$$

where F_z is the estimated flux at a greater depth (in this case 200 m), F_{zo} is the flux at a shallower reference depth (this is the POC flux collected at 100 m using the short-term drifting traps), z is the deep depth (200 m), z_o is the reference depth (100 m), and b is the attenuation coefficient that characterizes the shape of the vertical POC flux between the two depths.

Martin et al.[3] suggested a global attenuation coefficient (b = 0.858) based on POC flux measurements in the northeast Pacific. This b-value has been used in global models (e.g. [4]) and is influenced by aggregate settling velocities and degradation by both microbes and zooplankton[5–7]. A recent study suggested a fairly high b-value of 1.51 for Arctic waters with an Atlantic influence[8]. We have there used the low and high b-values suggested by Martin et al.[3] and Wiedmann et al.[8] to estimate the flux attenuation between 100 m and 200 m in order to calculate what the POC flux to 200 m would be based on the POC flux collected at 100 m during our research expedition (Supplementary Table 1). This resulted in an estimated POC flux to 200 m between 45 and 70 mg POC m^{-2} d^{-1} for the ice-covered region and between 22 and 35 mg POC m^{-2} d^{-1} for the ice-free region. This suggests that the estimate POC flux to 200 m for the ice-covered region are slightly higher than the long-term observations of POC flux during the ice-covered season (March, April and May, Fig. 1). The same is true for the estimated POC flux to 200 m for the ice-free region, when compared to the long-term observations for POC flux to 200 m during the ice-free season (June, July and August, Fig. 1). However, these small differences between estimated and collected POC flux at 200 m could also be due to differences in trap shapes, i.e. cylindrical versus conical shaped traps[9]. This suggests that the summer conditions at contrasting stations are, to some extent, representative of the changing export flux as affected by the seasonal cycle of sea ice. However, the flux mechanisms between the contrasting ice conditions should still be viewed as a proxy for potential export mechanisms during changing seasonal ice conditions, but seasonal and long-term changes in the Arctic are more complex due to changing plankton

composition, phenology of pelagic and sympagic algae as well as seasonally changing oceanographic features.

Supplementary Figures

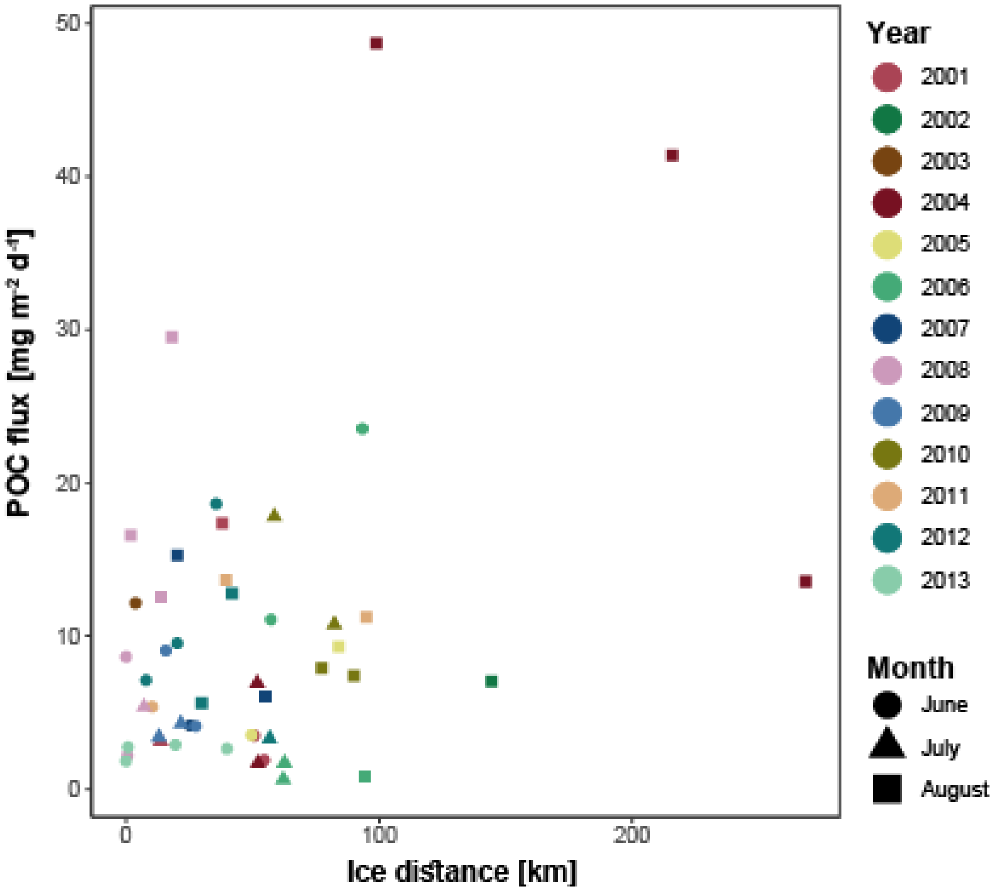

Supplementary Figure 1: Particulate organic carbon (POC) flux collected at 200 m by long-term moored sediment traps during summer flux peaks (June, July and August) of 2001-2013 plotted as a function of the distance to the ice-edge. The POC flux and the distance to the ice edge did not reveal significant relationship.

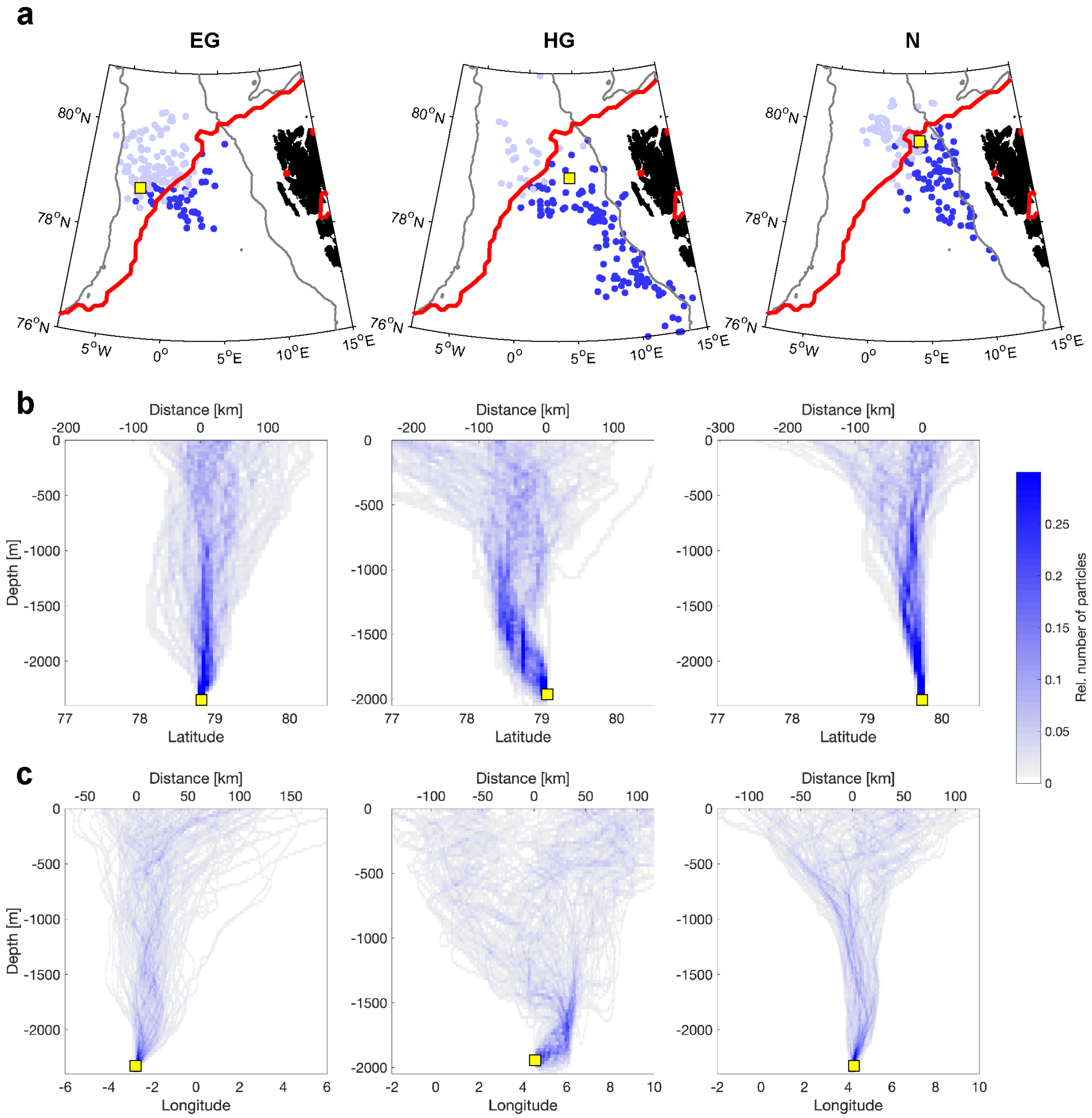

Supplementary Figure 2: Sinking trajectories of particles across the Fram Strait. Distribution of particle origin in surface (A), and sinking trajectories as a function of latitude (B) and longitude (C). The backward particle trajectories were computed for the time period March-July 2016, based on measured on board sinking velocities in ice-covered and ice-free regions (52 and 29 m d^{-1}, respectively). The yellow square indicates the starting point of the backward particle trajectory calculation. In panel A light blue dots represent aggregates originated in ice-covered waters, and dark blue dots represent aggregates originated in ice-free waters. The red line represents 15% ice-concentration during June-July 2016, and the gray line represents the 1000 m isobaths.

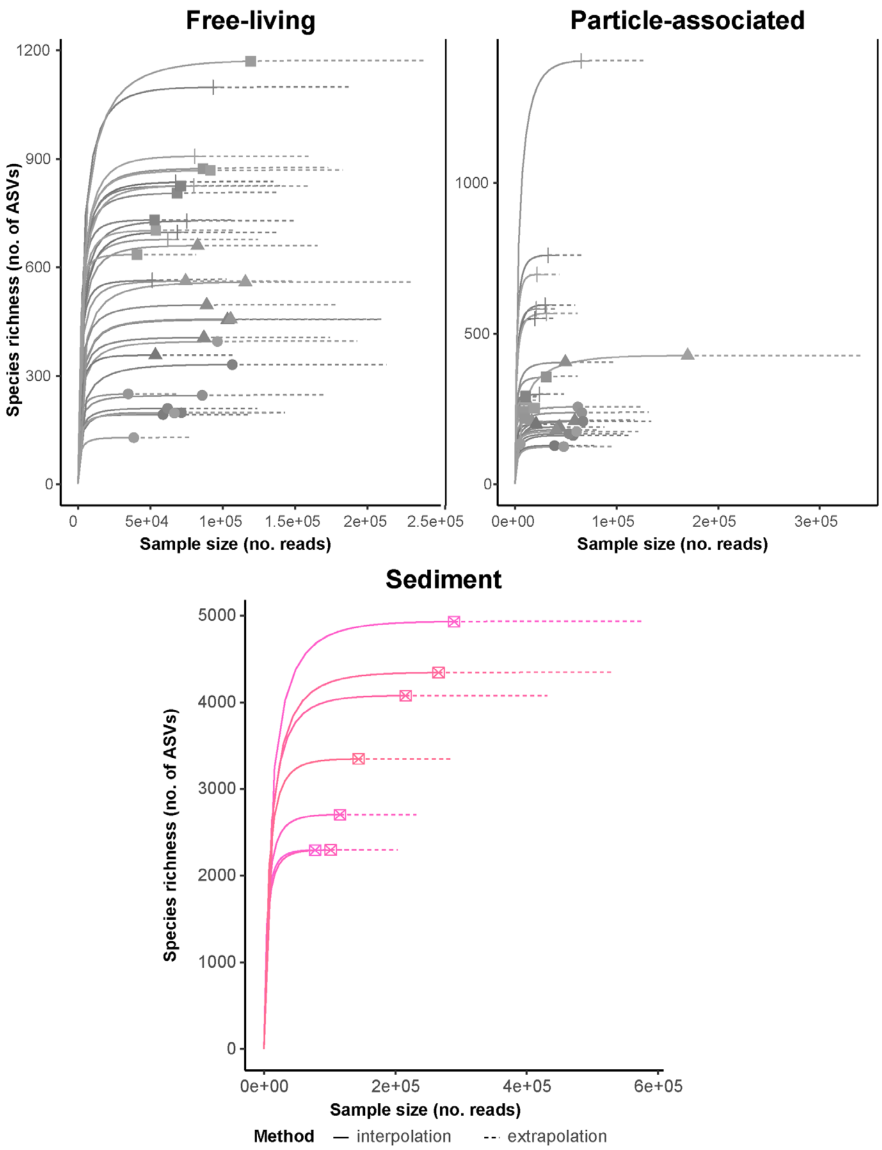

Supplementary Figure 3: Rarefactions of 16S rRNA gene analysis of bacterial and archaeal communities. The solid lines represent the observed accumulation with the number of reads sampled, and the dashed lines represent the extrapolated accumulation up to the double amount of reads. The observed values for each community are denoted by solid shapes. Sample-size-based rarefaction curves generated with the R-package "iNEXT", based on the Hill number of order q = 0. The rarefaction curves for each sample were generated based on 40 equally spaced rarefied sample sizes with 100 iterations.

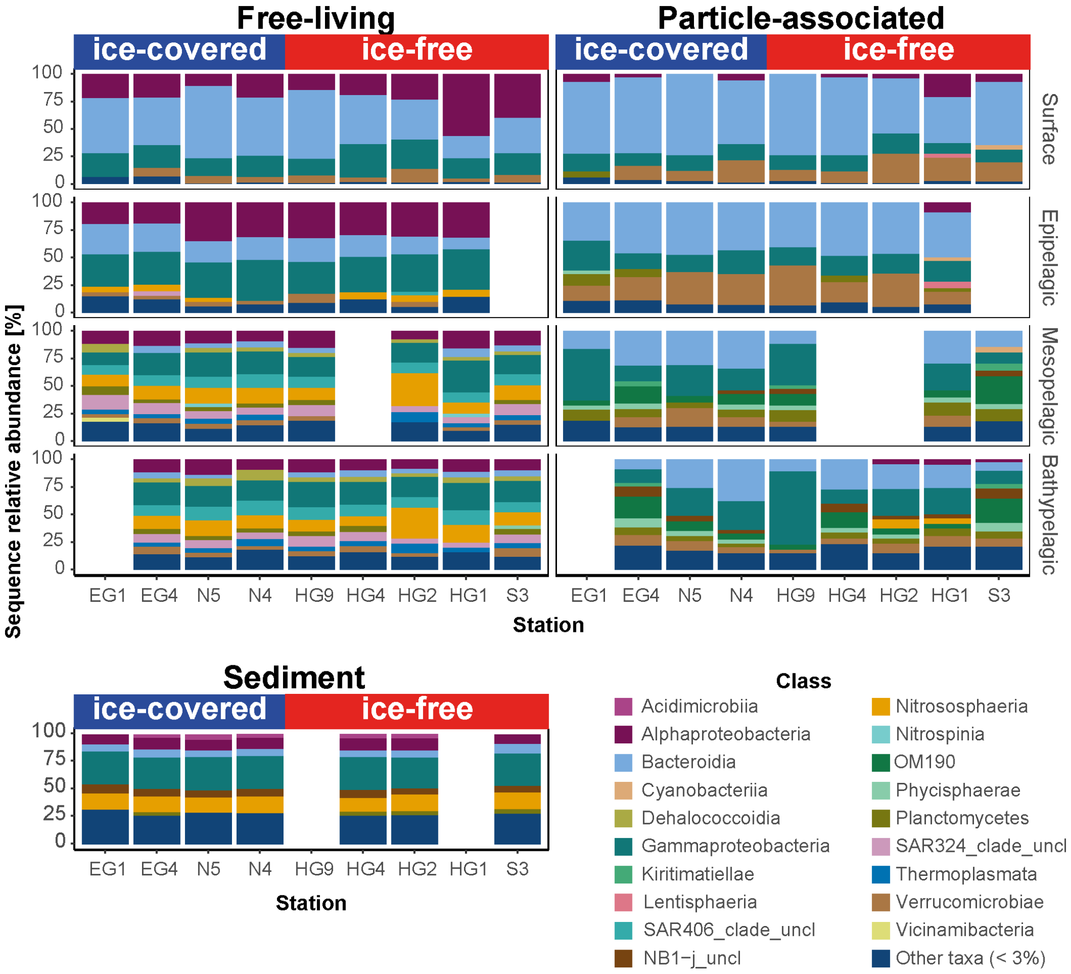

Supplementary Figure 4: Sequence proportion overview of bacterial and archaeal classes in free-living and particle-associated communities, and uppermost centimeter of deep-sea sediment. The classes represented by colors according to the legend, all classes with sequence proportion below 3% were classified as "Other classes".

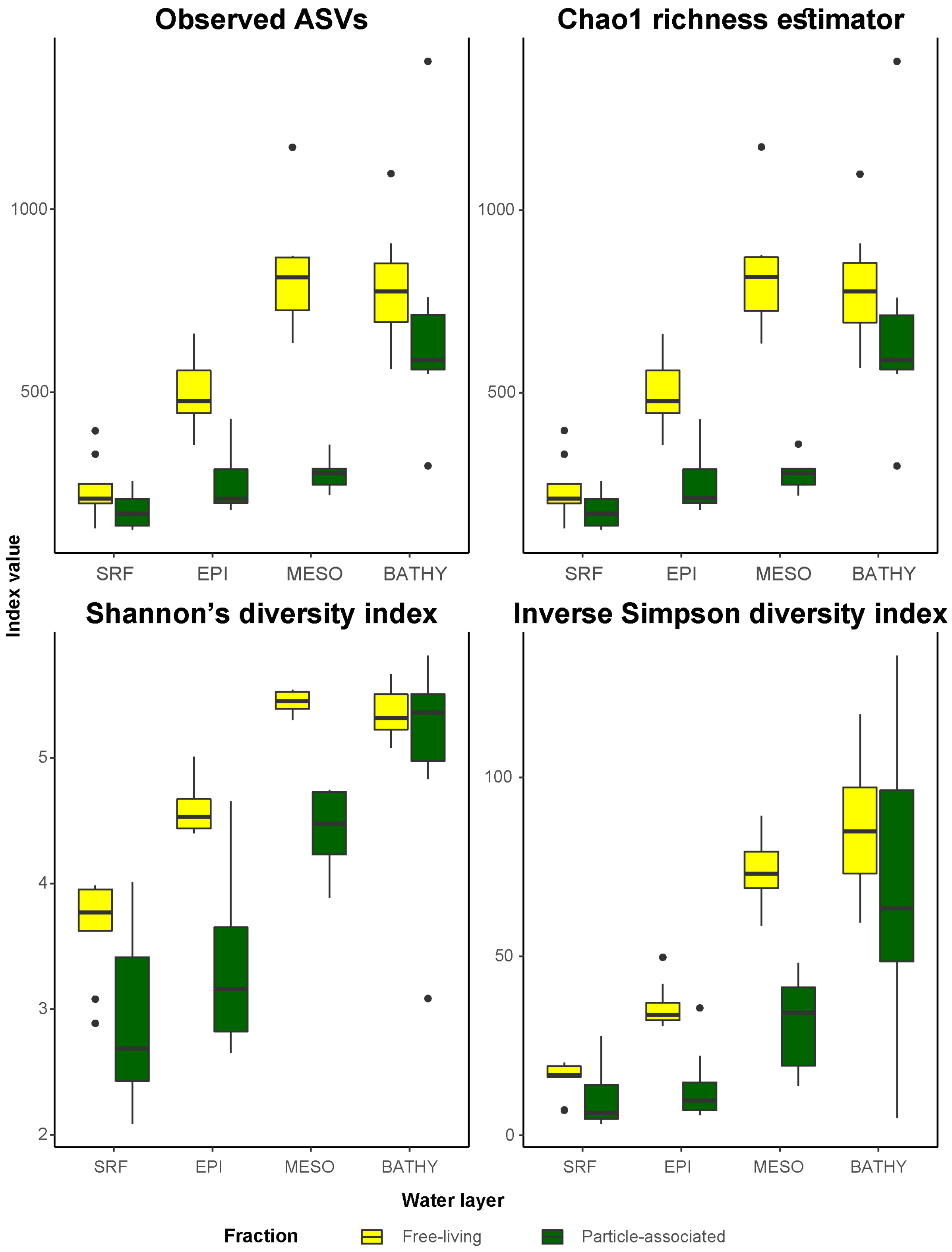

Supplementary Figure 5: Calculated alpha diversity indices of free-living and particle-associated bacterial and archaeal communities along the water column. The x-axis values represent the four distinct water layers: SRF - surface (10 - 30 m), EPI - epipelagic (100 m), MESO - mesopelagic (1000 m) and BATHY - bathypelagic (~50 m above the seafloor).

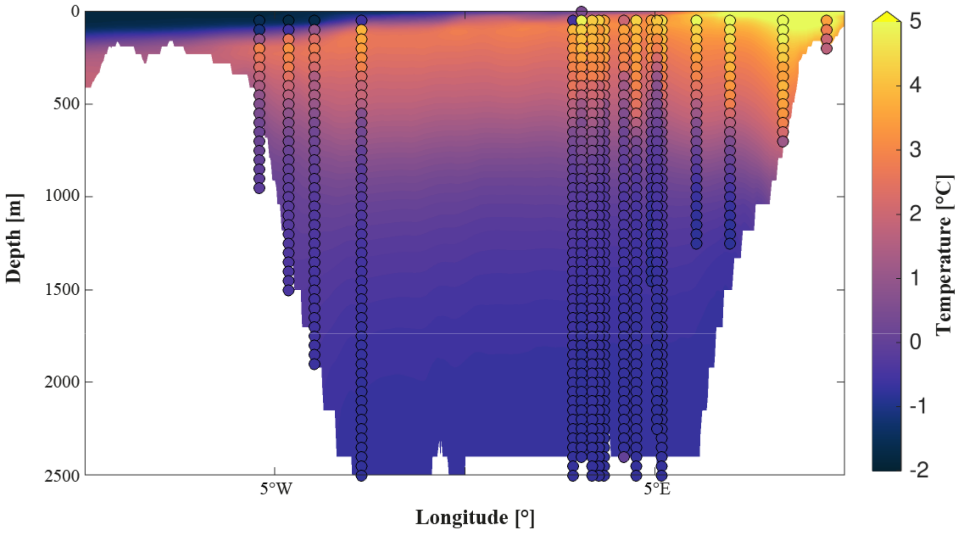

Supplementary Figure 6: Comparison of modeled and *in situ* measured water temperature across the Fram Strait. The background represents the mean model results for July 2016, which have been overlaid with the *in situ* measurements from the PS99.2 expedition. The division between warm Atlantic Water (AW) in the eastern Fram Strait and cold Arctic water in the western Fram Strait, the intermediate mixed Atlantic Water, and the cold Eurasian Basin Deep Water at bathypelagic depths, are clear in both the modeled results and the *in situ* measurements.

Supplementary References

1. Leu, E., Søreide, J. E., Hessen, D. O., Falk-Petersen, S. & Berge, J. Consequences of changing sea-ice cover for primary and secondary producers in the European Arctic shelf seas: Timing, quantity, and quality. *Prog. Oceanogr.* **90**, 18–32 (2011).

2. Tedesco, L., Vichi, M. & Scoccimarro, E. Sea-ice algal phenology in a warmer Arctic. *Sci. Adv.* **5**, eaav4830 (2019).

3. Martin, J. H., Knauer, G. A., Karl, D. M. & Broenkow, W. W. VERTEX: carbon cycling in the northeast Pacific. *Deep Sea Res. Part A. Oceanogr. Res. Pap.* **34**, 267–285 (1987).

4. Henson, S. A., Sanders, R. & Madsen, E. Global patterns in efficiency of particulate organic carbon export and transfer to the deep ocean. *Global Biogeochem. Cycles* **26**, n/a-n/a (2012).

5. Iversen, M. H. & Ploug, H. Ballast minerals and the sinking carbon flux in the ocean: carbon-specific respiration rates and sinking velocity of marine snow aggregates. *Biogeosciences* **7**, 2613–2624 (2010).

6. Ploug, H., Iversen, M. H., Koski, M. & Buitenhuis, E. T. Production, oxygen respiration rates, and sinking velocity of copepod fecal pellets: Direct measurements of ballasting by opal and calcite. *Limnol. Oceanogr.* **53**, 469–476 (2008).

7. Iversen, M. H. & Ploug, H. Temperature effects on carbon-specific respiration rate and sinking velocity of diatom aggregates – potential implications for deep ocean export processes. *Biogeosciences* **10**, 4073–4085 (2013).

8. Wiedmann, I. *et al.* Arctic Observations Identify Phytoplankton Community Composition as Driver of Carbon Flux Attenuation. *Geophys. Res. Lett.* **47**, (2020).

9. Baker, C. A., Estapa, M. L., Iversen, M., Lampitt, R. & Buesseler, K. Are all sediment traps created equal? An intercomparison study of carbon export methodologies at the PAP-SO site. *Prog. Oceanogr.* **184**, 102317 (2020).
