# Supplementary material for: Sea ice presence is linked to higher carbon export and vertical microbial connectivity in the Eurasian Arctic Ocean
Source: Commun Biol. 2021 Nov 3;4:1255. doi: 10.1038/s42003-021-02776-w (PMC8566512; doi:10.1038/s42003-021-02776-w)
Supplement: Supplementary file 2 — Description of Additional Supplementary Files [file 42003_2021_2776_MOESM2_ESM.pdf]

Description of Additional Supplementary Data Files

File name: Supplementary Data 1
Description: Microscopy counts of protist communities and estimated consumed inorganic nutrients across the Fram Strait.

File name: Supplementary Data 2
Description: Overview of microbial samples collected during RV Polarstern expedition PS99.2.

File name: Supplementary Data 3
Description: Estimated sources contribution to PA communities by 'SourceTracker'.

File name: Supplementary Data 4
Description: List of taxonomic groups with significantly enriched ASVs with depth along the water column.

File name: Supplementary Data 5
Description: Source data underlying the graphs and charts presented in the main figures.
