# Supplementary material for: Sea ice presence is linked to higher carbon export and vertical microbial connectivity in the Eurasian Arctic Ocean
Source: Commun Biol. 2021 Nov 3;4:1255. doi: 10.1038/s42003-021-02776-w (PMC8566512; doi:10.1038/s42003-021-02776-w)
Supplement: Supplementary file 8 — Reporting Summary [file 42003_2021_2776_MOESM8_ESM.pdf]

Corresponding author(s): Eduard Fadeev, Morten H. Iversen

Last updated by author(s): Dec 1, 2020

Reporting Summary

Nature Research wishes to improve the reproducibility of the work that we publish. This form provides structure for consistency and transparency in reporting. For further information on Nature Research policies, see our Editorial Policies and the Editorial Policy Checklist.

Statistics

For all statistical analyses, confirm that the following items are present in the figure legend, table legend, main text, or Methods section.

n/a	Confirmed	
☐	☒	The exact sample size (*n*) for each experimental group/condition, given as a discrete number and unit of measurement
☐	☒	A statement on whether measurements were taken from distinct samples or whether the same sample was measured repeatedly
☐	☒	The statistical test(s) used AND whether they are one- or two-sided<br>*Only common tests should be described solely by name; describe more complex techniques in the Methods section.*
☐	☒	A description of all covariates tested
☐	☒	A description of any assumptions or corrections, such as tests of normality and adjustment for multiple comparisons
☐	☒	A full description of the statistical parameters including central tendency (e.g. means) or other basic estimates (e.g. regression coefficient) AND variation (e.g. standard deviation) or associated estimates of uncertainty (e.g. confidence intervals)
☐	☒	For null hypothesis testing, the test statistic (e.g. *F*, *t*, *r*) with confidence intervals, effect sizes, degrees of freedom and *P* value noted<br>*Give P values as exact values whenever suitable.*
☐	☒	For Bayesian analysis, information on the choice of priors and Markov chain Monte Carlo settings
☒	☐	For hierarchical and complex designs, identification of the appropriate level for tests and full reporting of outcomes
☐	☒	Estimates of effect sizes (e.g. Cohen's *d*, Pearson's *r*), indicating how they were calculated

Our web collection on statistics for biologists contains articles on many of the points above.

Software and code

Policy information about availability of computer code

Data collection	16S amlicon sequencing was performed on an Illumina MiSeq platform.
Data analysis	Primer trimming using cutadapt (v1.9), Martin 2011<br>Taxonomy assignment using Silva reference database, release 138<br>R (v3.6.3) and Rstudio (v1.2.5033)<br>R packages:<br>'dada2' (v1.14.1), Callahan et al. 2016<br>'phyloseq' (v1.28.0),McMurdie and Holmes, 2013<br>'ggplot2' (v3.3.0), Gómez-Rubio, 2017<br>'iNEXT' (v2.0.20), Hsieh et al., 2018<br>'DEseq2' (v1.24.0), Love et al., 2014<br>'SourceTracker' (v1.0), Knights et al., 2011<br><br>The custom code that was used for the statistical analyses is deposited on GitHub dedicated repository that will become public once the manuscript is published (or earlier, upon your request).

For manuscripts utilizing custom algorithms or software that are central to the research but not yet described in published literature, software must be made available to editors and reviewers. We strongly encourage code deposition in a community repository (e.g. GitHub). See the Nature Research guidelines for submitting code & software for further information.

Data

Policy information about availability of data

All manuscripts must include a data availability statement. This statement should provide the following information, where applicable:

- Accession codes, unique identifiers, or web links for publicly available datasets
- A list of figures that have associated raw data
- A description of any restrictions on data availability

The raw, primer trimmed paired-end sequencing reads were deposited in the European Nucleotide Archive (ENA) under accession number PRJEB30254. All the environmental data used in this study is available in the manuscript and its supplementary material.
The raw environmental data is available on PANGAEA data publisher:
Hydrographic data of the seawater including temperature and salinity - doi:10.1594/PANGAEA.871952
Inorganic nutrient concentrations - doi:10.1594/PANGAEA.906132
Long-term sediment trap data - doi: 10.1594/PANGAEA.855473

Field-specific reporting

Please select the one below that is the best fit for your research. If you are not sure, read the appropriate sections before making your selection.

☐ Life sciences ☐ Behavioural & social sciences ☒ Ecological, evolutionary & environmental sciences

For a reference copy of the document with all sections, see nature.com/documents/nr-reporting-summary-flat.pdf

Ecological, evolutionary & environmental sciences study design

All studies must disclose on these points even when the disclosure is negative.

Study description	To investigate the effect of sea-ice on vertical export in Arctic waters, we assessed sinking marine aggregates composition using biochemical and microscopy methods, and characterized the microbial communities associated with them and the surrounding waters using 16S amplicon sequencing. The V4-V5 variable region of the 16S rRNA was amplified and subsequently sequenced on an Illumina MiSeq platform.
Research sample	The sampling area of the Fram Strait is characterized by ice-covered and ice-free regions during the summer. For the molecular analsis we sampled 4 stations in the ice-covered part of the Strait, and 5 stations in the ice-free part. The marine aggregtes in situ sampling consisted of 2 stations in the ice-covered region and 2 stations in the ice-free region.
Sampling strategy	A CTD rosette was used to collect water samples from 4 different water depths were sampled, from surface down to the deep ocean, and each water sample was size fractionated for free-living (0.2-5 um) and particle-associated (>5 um) microbial communities.
Data collection	Marine aggregates were collected and characterized by MHI, according to existing protocols by Ploug and Jørgensen, 1999 and Thiele at al., 2015.<br>Phytoplankton cell counts were conducted on fixed water samples according to existing protocols by Utermöhl, 1958, and Edler, 1979. (EMN)<br>Genomic DNA was extracted, amplified and sequenced on Illumna MiSeq platform (EF).
Timing and spatial scale	All samples were collected in the Fram Strait (79° N, 0°E)during the research expedition PS99.2 with RV Polarstern, between June 24th and July 16th 2016. The exact sampling time for each station is stated in the supplementary material of the manuscript.
Data exclusions	Sequences that were taxonomically unclassified on domain level, or not assigned to bacterial or archaeal lineages, were excluded from further analysis. Furthermore, all sequences which were taxonomically assigned to mitochondria and chloroplast were removed from the dataset.
Reproducibility	All sequencing of the free-living and particle-associated communities was conducted in technical duplicates of each biological samples, which yielded similar results. All necessary protocols and laboratory procedures to reproduce 16S analysis are mentioned in the manuscript, along with references to all applied software and codes.
Randomization	Randomization was relevant only in the microbial source tracking analysis, where it was conducted using subsampling of the microbial communities.
Blinding	Blinding was relevant only in the microbial source tracking analysis, where it was conducted using a leave-one-out (LOO) approach.

Did the study involve field work? ☒ Yes ☐ No

Field work, collection and transport

Field conditions	Ship-based sampling was generally conducted during calm weather conditions. The relevant oceanographic conditions for each sample are described in the supplementary material of the manuscript.
Location	The study was conducted in the Fram Strait (79°N,0°E). The exact locations of each sampled station are described in the

	supplementary material of the manuscript.
Access & import/export	All field work was conducted in compliance with national and international regulations and necessary authorization was granted by the german enviromental agency.
Disturbance	No particular disturbance was caused during sampling.

Reporting for specific materials, systems and methods

We require information from authors about some types of materials, experimental systems and methods used in many studies. Here, indicate whether each material, system or method listed is relevant to your study. If you are not sure if a list item applies to your research, read the appropriate section before selecting a response.

Materials & experimental systems

n/a	Involved in the study
☒	☐ Antibodies
☒	☐ Eukaryotic cell lines
☒	☐ Palaeontology and archaeology
☒	☐ Animals and other organisms
☒	☐ Human research participants
☒	☐ Clinical data
☒	☐ Dual use research of concern

Methods

n/a	Involved in the study
☒	☐ ChIP-seq
☒	☐ Flow cytometry
☒	☐ MRI-based neuroimaging
